# Nootropic benzothiazoles promote dendritic spine formation by targeting fascin-1

**DOI:** 10.1016/j.jbc.2025.110572

**Published:** 2025-08-08

**Authors:** Aashish Shivkumar, Kyle R. Berg, Kevin C. Sibucao, Geoffray Leriche, Lara E. Dozier, Carla A. Espinoza, Gentry N. Patrick, Zied Gaieb, Christian Seitz, Rommie E. Amaro, Hyun-Hee Park, Hyang-Sook Hoe, Jacob Wozniak, David J. Gonzalez, Saptarshi Sinha, Pradipta Ghosh, Jerry Yang

**Affiliations:** 1 Department of Chemistry and Biochemistry, University of California, San Diego, La Jolla, California, USA; 2 The Section of Neurobiology in the Division of Biological Sciences, University of California, San Diego, La Jolla, California, USA; 3 Department of Neural Development and Disease, Korea Brain Research Institute (KBRI), Daegu, South Korea; 4 Department of Pharmacology and the Skaggs School of Pharmacy and Pharmaceutical Sciences, University of California, San Diego, La Jolla, California, USA; 5 Department of Cellular and Molecular Medicine, University of California San Diego, La Jolla, California, USA; 6 Department of Medicine, University of California San Diego, La Jolla, California, USA

**Keywords:** Alzheimer’s disease, dendritic spine, benzothiazoles, neuron, fascin, actin, molecular docking, mutagenesis

## Abstract

Identifying new drug targets to enhance memory and learning is essential for treating neurodegenerative diseases, such as Alzheimer’s disease. We previously showed that benzothiazole amphiphiles can improve memory and learning by increasing dendritic spine and synaptic density in both WT mice and in a transgenic Alzheimer’s model mice. The cellular target for this class of compounds, however, was unknown. Using a photoaffinity-labeling approach, we identify fascin-1 as the major protein target in neurons for these compounds. These compounds enhance spine density by directly modulating actin dynamics, increasing the capability of fascin-1 to bundle actin filaments. Molecular docking and structure-guided mutagenesis studies reveal a distinct binding site on fascin-1, differing in both location and function from previously reported fascin-1-targeting molecules, opening exciting new avenues for selectively tuning fascin-1 activity in the brain.

Alzheimer’s disease (AD) and related forms of dementia are becoming more prevalent as the average human lifespan increases (1). In particular, neurodegenerative diseases (NDDs), such as AD, have been diagnosed in alarming numbers globally. Decades of research focused on the amyloid cascade hypothesis have only very recently led to the development of disease-modulating antibody-based therapeutics (2, 3), although their efficacy for improving memory or slowing cognitive decline in AD still needs improvement (4). Consequently, there is a pressing need for new strategies for the development of therapeutics to treat AD and other memory impairment disorders. For instance, synapse loss/dysfunction is a hallmark feature of many NDDs (5, 6, 7, 8) and is believed to be among the earliest phenotypic events in the progression of AD. A method that can promote the creation and maintenance of synapses could, therefore, represent an effective pathway for the treatment of AD and potentially other conditions that are accompanied by memory loss.

The excitatory synapse created between neuronal axonal inputs and postsynaptic dendritic spines is considered a fundamental structural cellular feature in memory formation and storage (9). Previous evidence supports a strong positive correlation between the density of hippocampal dendritic spines and memory and learning (10, 11). Dendritic complexity, synaptogenesis, and overall proper development and function of neurons are regulated by growth factors, such as brain-derived neurotrophic factor (12). While some small molecules have been reported to exhibit neurotrophin-like activity with respect to promoting neuritic outgrowth, none of these molecules promote dendritic spine formation (13, 14, 15). To date, relatively few small-molecule compounds are known to increase dendritic spine density (16, 17, 18, 19, 20). Among these molecules are oligo (ethylene glycol) derivatives of benzothiazole aniline (BTA), of which we have previously shown that the tetra (ethylene glycol) derivative BTA-EG_4_ improves memory and learning in both WT mice and a transgenic mouse model for AD (21). These behavioral improvements in treated mice were accompanied by significant increases in dendritic spine and synaptic density in hippocampal neurons. Other BTA derivatives, such as BTA-EG_6_, BAM1-EG_6_, BAM2-EG_6_, and BAM3-EG_6,_ show similar phenotypic activity on spine density increases in primary neurons (16). The molecular target and mechanism leading to this phenotypic activity by these compounds, however, have not been reported. Identification of this target could reveal a novel pathway for treating AD and other NDDs.

Here, we designed a photoaffinity analog of BTA-EG_4_, compound 1 (Fig. 1*A*), which we found to be highly selective for covalently labeling fascin-1 in cellular and brain lysates. A major function of fascin-1 is to enable bundling of actin filaments and facilitate the formation of filipodia in neurons (22), hence playing a central role in regulating actin dynamics. We show that knockdown of fascin-1 expression levels in primary neurons leads to a decrease in spine density that could not be rescued by the BTA compounds, linking the binding of this protein to the phenotypic effects of the small molecules on spine dynamics in neurons. We also demonstrate that BTA-EG_4_ and BTA-EG_6_ increase the actin-bundling activity of fascin-1 and use molecular docking–guided mutagenesis studies to identify a novel binding site for these molecules on fascin-1.

**Figure 1:**
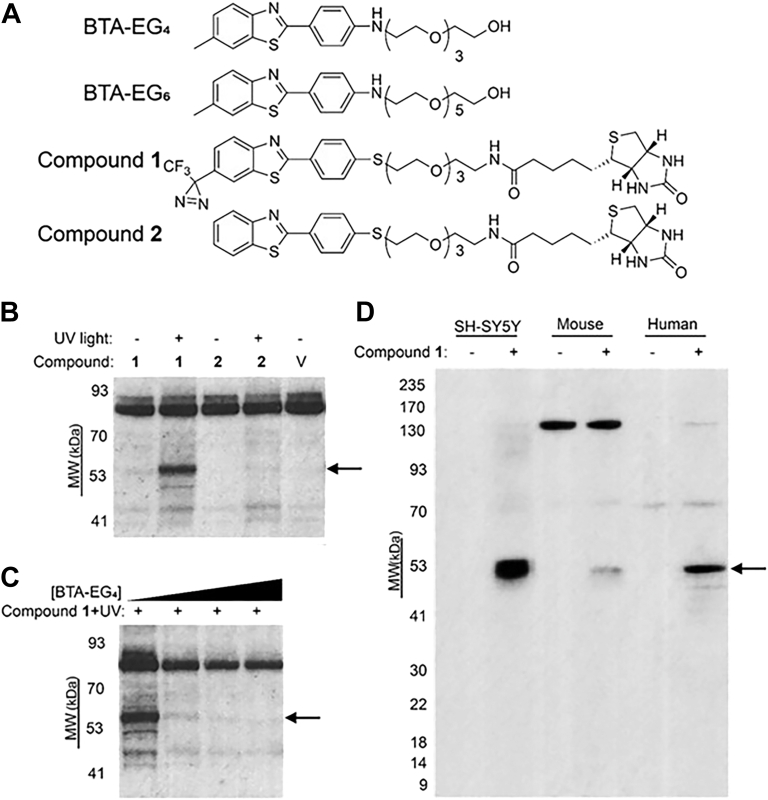
**Photoaffinity-labeling studies of an analog of BTA-EG_4_ in cell and brain lysates.***A*, structure of benzothiazole-based compounds used in this study. *B*, Western blot of photoaffinity-labeling experiments in human brain cortex lysate using compounds 1 and 2 in the presence or absence of UV light for photoactivation. Blots were visualized using a streptavidin–HRP conjugate, bands found across all samples or in negative control lanes (in *B*–*D*) are presumed to be natively biotinylated proteins. *C*, photoaffinity-labeling experiments using compound 1 in human brain cortex lysate with increasing concentrations of BTA-EG_4_ (0–500 μM). Uncropped versions of Figure 1, *B* and *C* are provided as Figs. S2, *A* and *B*. *D*, crosslinking experiment done with lysates from SH-SY5Y neuroblastoma, APP/PS1 mouse midbrain, and human brain cortex in the presence or absence of compound 1. The *black arrows* indicate the ∼53 kDa band corresponding to the protein strongly photoaffinity labeled by compound 1. BTA, benzothiazole aniline.

## Results

### Photoaffinity-labeling studies

In order to identify the cellular target for BTA-EG_4_, we synthesized an analog of BTA-EG_4_ (compound 1, Fig. 1*A*) carrying a trifluoromethyldiazirine group that can be used to crosslink the molecule to protein targets upon exposure to UV light (23, 24) (see Supplementary Note S1 and Fig. S1 for the synthesis and photo reactivity studies of compound 1). Compound 1 also contains a biotin tag for identification and retrieval of proteins after photoaffinity labeling. Since biotinylated compounds typically lack cell permeability because of their large size and hydrophilicity (25, 26, 27), compound 1 was used solely for target identification in lysates and was not tested for bioactivity in living neurons. We incubated compound 1 with lysates from human brain cortex and exposed to UV light. Western blot analysis of this crude mixture using a streptavidin–horseradish peroxidase (HRP) conjugate revealed several bands of natively biotinylated proteins and a new strong band around 53 kDa that was only present in lysates containing compound 1 after exposure to UV light (Fig. 1*B*). Performing the same experiment with vehicle control and compound 1 without UV exposure, or compound 2, which lacked the photoreactive trifluoromethyldiazirine group, did not result in capturing this 53 kDa band of protein (Fig. 1*B*). Increasing concentrations of BTA-EG_4_ effectively inhibited photoaffinity labeling of proteins with compound 1, supporting that compound 1 and BTA-EG_4_ target the same protein in human brain cortex lysate (Fig. 1*C*).

A consistently strong band was visualized at ∼53 kDa for the photoaffinity-labeling experiment with compound 1 performed with lysates from SH-SY5Y neuroblastoma or lysates from APP/PS1 mouse midbrain (an AD mouse model (28)) or human brain cortex (Fig. 1*D*). This band was not observed in any of the lysate samples when the same procedure was performed in the absence of compound 1. We also observed gel bands around 130 kDa and 80 kDa that were present in the Western blot analysis of mouse brain and human cortex lysates exposed to UV light in the presence or absence of compound 1, which we attributed to natively biotinylated proteins in these samples (Fig. 1*D*). Extraction and tandem mass spectrometry (MS) analysis of the ∼53 kDa band of protein photolabeled with compound 1 from SH-SY5Y neuroblastoma lysates revealed fascin-1 (54.5 kDa) as the major protein target and was confirmed by Western blot analysis (Fig. S2 and Table S1).

### Knockdown of fascin-1 expression levels in neurons decreases dendritic spine density

**Figure 2:**
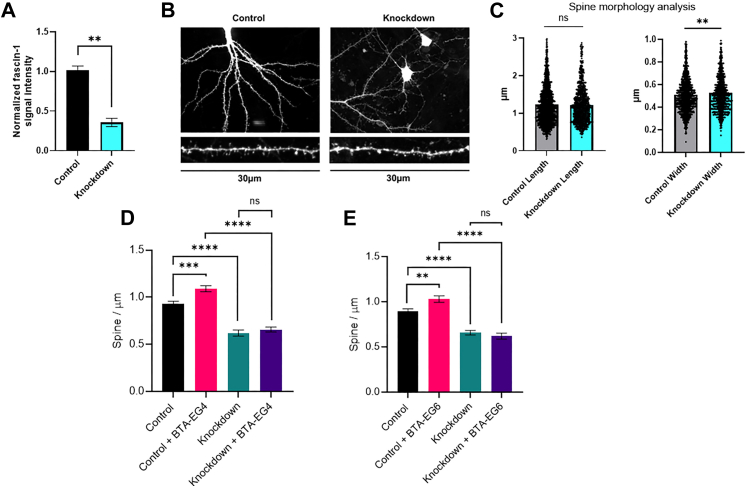
**Analysis of fascin-1 expression in PC12 cells and spine density and morphology measurements upon treatment with plasmids in primary hippocampal neurons.***A*, quantification of fascin-1 expression levels in PC12 cells treated with knockdown AAV plasmid. *B*, representative 30 μm dendritic segments in primary hippocampal neurons selected for dendritic spine analysis. *C*, spine morphology analysis of primary neurons after fascin-1 knockdown. Statistical analysis was done using two-tailed Welch’s *t* test (∗∗*p* < 0.01). *D*, spine density measurements in primary neurons upon treatment with AAV vectors in the presence of vehicle control (0.1% DMSO) and BTA-EG_4_ (5 μM). *E*, spine density measurements in primary neurons upon treatment with AAV vectors in the presence of vehicle control (0.1% DMSO) and BTA-EG_6_ (5 μM). Statistical analysis was performed using two-tailed Welch’s *t* test (∗∗*p* < 0.1, ∗∗∗*p* < 0.01, and ∗∗∗∗*p* < 0.001). *N* = 60 dendritic segments per treatment. AAV, adeno-associated virus; BTA, benzothiazole aniline; DMSO, dimethyl sulfoxide; HRP, horseradish peroxidase.

After identifying fascin-1 as the target of BTA-EG_4,_ we examined the effect of knocking down fascin-1 expression levels on spine morphology and density in primary hippocampal neurons. For fascin-1 knockdown, we designed adeno-associated virus (AAV) vector that we showed could reduce fascin-1 expression levels by 65% (Figs. 2*A* and S3) in PC12 cells (a rodent neuroblastoma cell line). We also found that primary neurons infected with this shRNA AAV exhibited a ∼60% reduction in fascin-1 expression compared with scramble control (Fig. S3).

To examine the effects of fascin-1 knockdown on spine morphology and density in primary hippocampal neurons compared with control cells, we performed a blinded analysis of 30 μm dendritic segments (Fig. 2*B*) from a random sampling of 60 neurons (20 neurons per replicate) in each condition to estimate the number, length, and width of spines. We found a small but significant increase in the width of spines in cells with fascin-1 knockdown compared with control cells (Fig. 2*C*). However, no significant difference in spine length was observed in cells with fascin-1 knockdown compared with control cells (Fig. 2*C*). Our previous study showed that these benzothiazole amphiphiles do not significantly affect spine morphology (16).

We found that fascin-1 knockdown led to a significant decrease in spine density compared with cells with endogenous fascin-1 expression levels (Fig. 2, *D* and *E*). This result is in contrast to treatment of primary hippocampal neurons with 5 μM BTA-EG_4_ or BTA-EG_6_ (Fig. 2, *D* and *E*), which led to significant increases in spine density, consistent with previous reports (16, 29, 30). We assessed whether BTA compounds could recover the phenotypic reduction seen in spine density with fascin-1 knockdown plasmid and found that BTA compounds were unable to increase spine density when fascin-1 is knocked down (Fig. 2, *D* and *E*). This result strongly supports that the binding of BTA compounds to fascin-1 is important for the phenotypic increase in dendritic spine density of neurons seen when exposed to these molecules.

Immunostaining of endogenous fascin-1 in a mature primary neuron (DIV21) revealed that fascin-1 was not only located throughout the cytosol but also localized in dendritic protrusions that are consistent with dendritic spines (Fig. S4). We also found that treatment of primary neurons with 5 μM BTA-EG_4_ or BTA-EG_6_ did not significantly alter fascin-1 expression levels (Fig. S5), suggesting that these molecules induce the phenotypic activity of increasing dendritic spine density by directly impacting the dynamic function of endogenous fascin-1 rather than by influencing a proteome-level change in expression.

### BTA compounds increase actin bundling by fascin-1

**Figure 3:**
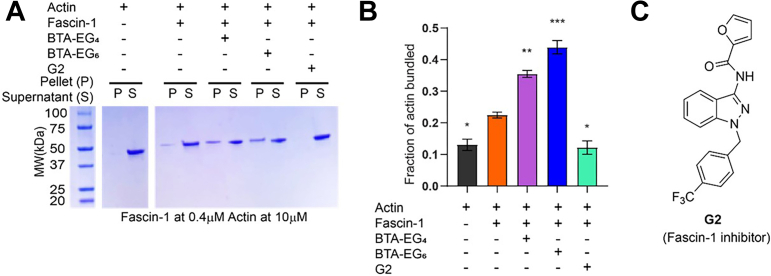
**Examination of the effects of fascin-1 ligands on the bundling of actin filaments.***A*, representative gel of actin in bundles (P) *versus* unbundled (S). The concentration of fascin-1 and actin remained constant at 0.4 μM and 10 μM, respectively. Full gels are provided in Fig. S7. *B*, quantification of the gel in (*A*) showing the fraction of actin filaments in bundles after various treatments. All experiments contained a final concentration of 20 μM small molecule. Statistical analysis was done by two-tailed Welch’s *t* test (∗*p* < 0.05, ∗∗*p* < 0.01, and ∗∗∗*p* < 0.001) for N = 3 per condition. *C*, molecular structure of G2, a known inhibitor of fascin-1 bundling activity.

Since fascin-1 is known to bundle actin filaments, we assessed the effects of BTA compounds on the function of fascin-1 using a previously reported actin bundling and sedimentation assay (31). We found that both BTA-EG_4_ and BTA-EG_6_ significantly increase the actin-bundling capability of fascin-1 (Fig. 3, *A* and *B*). This result is in contrast to G2 (Fig. 3*C*), a known inhibitor of fascin-1 (31, 32), which effectively diminished actin bundling by fascin-1 (Fig. 3, *A* and *B*). These data suggest that, although both BTA compounds and G2 bind to fascin-1, they have an opposite effect on fascin-1 activity. We also found that the treatment of primary neurons with G2 does not lead to a significant change in spine density compared with control cells (Fig. S6), but the poor solubility and unknown cellular uptake of G2 could affect its activity in cells.

### BTA compounds alter fascin-1 interactions in brain lysates

**Figure 4:**
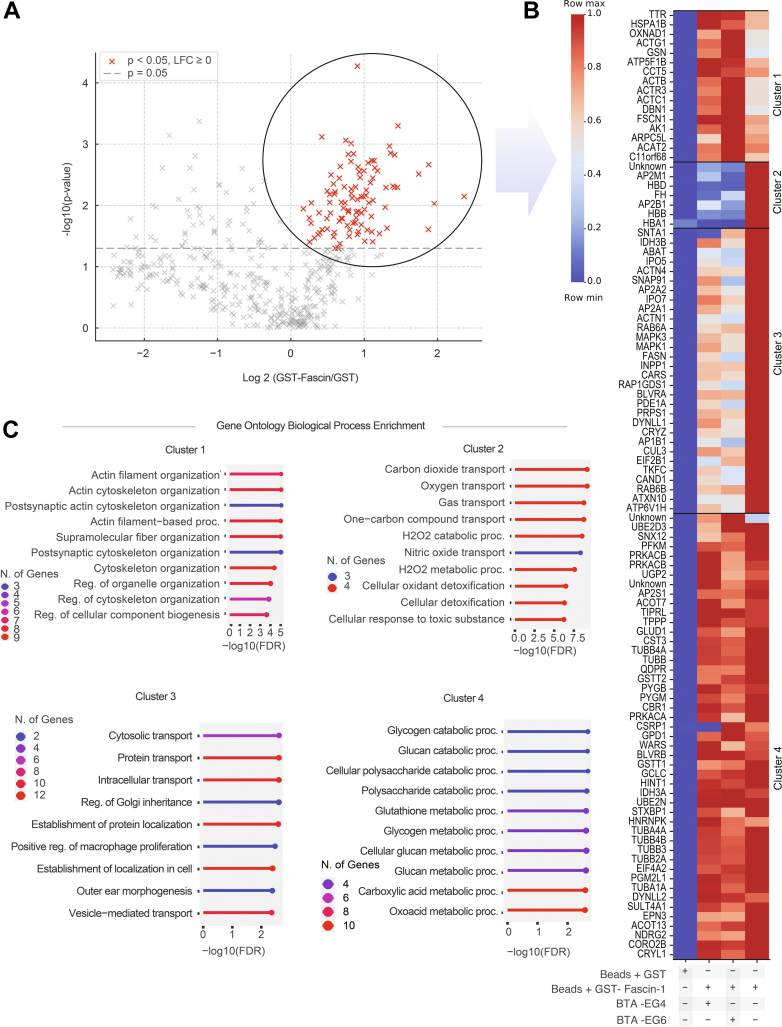
**TMT mass spectrometry analysis of fascin-1 pulldowns in the presence or absence of BTA-EG_4_ and BTA-EG_6_.***A*, volcano plot highlighting fascin-1 interactors identified in the pulldown experiment with *p* < 0.05. *B*, K-means clustered heat map of all significant fascin-1 interactors from the volcano plot. *Blue* indicates a low presence of protein pulled down, and *red* indicates a high presence of the protein pulled down compared with no BTA molecule present. Cluster 1 shows proteins with increased interaction with fascin-1 in the presence of BTA-EG_4_ and BTA-EG_6_. Cluster 2 shows proteins with complete dissociation from fascin-1 in the presence of BTA-EG_4_ and BTA-EG_6_ relative to negative control. Cluster 3 shows proteins with moderately decreased interaction with fascin-1 in the presence of BTA-EG_4_ and BTA-EG_6_. Cluster 4 shows proteins whose affinity for fascin-1 remains unchanged in the presence of BTA-EG_4_ and BTA-EG_6_. (N = 3 for each condition). *C*, Gene Ontology enrichment analysis of proteins, mapped to their corresponding genes, from each cluster revealed significantly associated biological processes (FDR <0.05). The color scale represents the number of genes contributing to each enriched term. BTA, benzothiazole aniline; FDR, false discovery rate; TMT, tandem mass tag.

To further examine whether BTA-EG_4_ and BTA-EG_6_ modulate actin dynamics, we performed a tandem mass tag (TMT) MS experiment to assess how these compounds affect fascin-1 interactions with cellular proteins in human brain cortex lysates. Using glutathione beads carrying a glutathione-*S*-transferase (GST)-fascin-1 fusion protein, we enriched for fascin-1-interacting proteins and compared them with a control pulldown using GST alone. This experiment identified 100 proteins that interact directly or indirectly with fascin-1 (Fig. 4*A*). The overall interaction profiles were similar between brain samples treated with BTA-EG_4_ and BTA-EG_6_ (Fig. 4*B*), indicating that both compounds modulate fascin-1 interactions in a consistent manner. Specifically, we identified 15 proteins with markedly increased pulldown (Fig. 4*B*, cluster 1), 8 proteins with substantially decreased pulldown (Fig. 4*B*, cluster 2), 28 proteins with moderately decreased pulldown (Fig. 4*B*, cluster 3), and 49 proteins with unchanged pulldown levels (Fig. 4*B*, cluster 4) with fascin-1 in the presence of BTA-EG_4_ and BTA-EG_6_ compared with no compound present.

Gene Ontology enrichment analysis of biological processes revealed that cluster 1 proteins, enriched in fascin-1 pulldown in the presence of BTA compounds, are primarily associated with actin filament organization (Fig. 4*C*). In contrast, clusters 2 and 3, which show varying degrees of depletion in the pulldown, do not exhibit enrichment in any specific biological processes. Proteins with no significant change in pulldown levels (cluster 4) are predominantly associated with diverse metabolic processes. These findings suggest that BTA-EG_4_ and BTA-EG_6_ selectively enhance the role of fascin-1 in actin organization, without broadly altering metabolic or housekeeping pathways.

Notably, cluster 1 included several isoforms of actin, as well as other known actin-binding proteins, such as gelsolin (33), drebrin (34), ARP3, and ARPC5L (a subunit of the ARP2/3 complex) (35). Conversely, cluster 2 proteins, whose interaction with fascin-1 was reduced, included α-actinins 1 and 4 (another class of actin-binding proteins (36)) and AP2 complex (a protein involved in clathrin-mediated endocytosis (37)) (see Supplementary Data File 1 for a full list of proteins identified from the TMT analysis).

Together, these results support that BTA compounds increase spine density in neurons by targeting fascin-1 and enhancing its ability to reorganize actin networks.

### Identification of the binding site for BTA-EG_6_ on fascin-1

**Figure 5:**
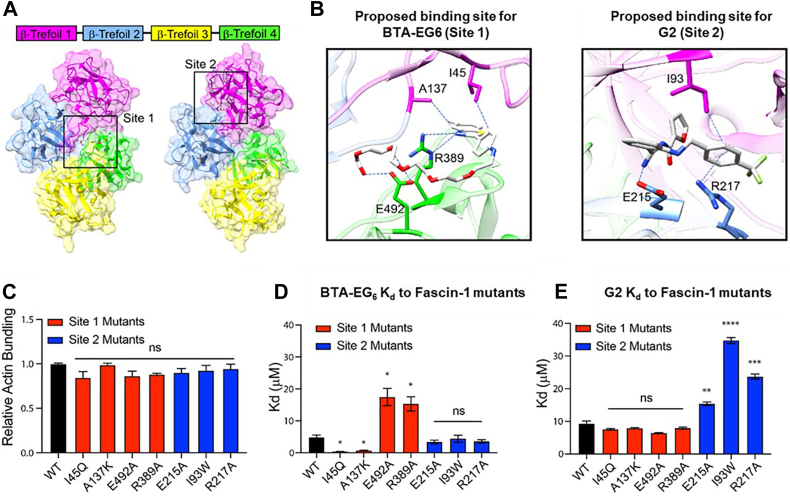
**Results from molecular docking and mutagenesis studies.***A*, two proposed binding sites (sites 1 and 2) on fascin-1 for small molecules were identified (*solid rectangle*). *B*, representative top docked poses of BTA-EG_6_ and G2 bound to fascin-1 at sites 1 and 2, respectively. *C*, analysis of the actin-bundling capability of WT fascin-1 and seven fascin-1 mutant proteins. *D*, *K*_*d*_ values of BTA-EG_6_ to WT fascin-1 and seven fascin-1 mutant proteins as estimated by ITC measurements. *E*, *K*_*d*_ values of G2 to WT fascin-1 and seven fascin-1 mutant proteins as estimated by ITC. Statistical analysis was performed using a two-tailed Welch’s *t* test (∗*p* < 0.05, ∗∗*p* < 0.01, ∗∗∗*p* < 0.001, and ∗∗∗∗*p* < 0.0001). N = 3 for each experiment. BTA, benzothiazole aniline; ITC, isothermal titration calorimetry.

We used isothermal titration calorimetry (ITC) to quantify and confirm that BTA-EG_6_ binds directly to recombinantly expressed WT fascin-1, with a 1:1 protein:ligand stoichiometry and a dissociation constant (*K*_*d*_) of approximately 5 μM (Fig. S8). Here, we chose to use BTA-EG_6_ as a representative example in this binding analysis because of its increased solubility in aqueous solution compared with BTA-EG_4_. The X-ray structure of fascin-1 has been reported by several groups, showing that this monomeric protein contains four β-trefoil domains, with domains 1/2 and 3/4 related by twofold pseudosymmetry (38, 39) (Fig. 5*A*). The actin-binding regions have been identified along β-trefoils 1 and 2, and 1 and 4, with a possible third actin-binding region located on β-trefoil 3 (40). While we have been able to obtain our own X-ray structure of recombinant fascin-1, attempts at cocrystallization of fascin-1 and a BTA ligand have not yet been successful. We, therefore, used *in silico* tools to investigate the potential binding pockets for BTA ligands on fascin-1. Schrodinger SiteMap (41) tool analyses revealed two probable binding sites (sites 1 and 2) for small molecules on the fascin-1 structure (Protein Data Bank ID: 1DFC (38)) (Fig. 5*A*). To obtain additional insight into these binding sites, we carried out molecular docking studies in which BTA-EG_6_ and G2 (fascin-1 inhibitor (31, 32)) were separately docked into both the binding sites obtained from SiteMap, for a total of four docking runs (Figs. 5*B* and S9; the Experimental procedures section summarizes the details of the docking studies). Site 1 (between β-trefoils 1 and 4) is adjacent to an actin binding site of fascin-1 and is in close proximity to Ser39 in β-trefoil 1 (a regulatory phosphorylation site on fascin-1) (42). Site 2 (between β-trefoils 1 and 2) has been reported as the binding site for G2 analogs (31).

For site 1, the top scoring poses of BTA-EG_6_ revealed molecular interactions between the benzothiazole group of BTA-EG_6_ and hydrophobic residues in fascin-1 such as A137 and I45, whereas the benzothiazole nitrogen forms a H-bond interaction with R389. Residue E492 is also predicted to be involved in multiple H-bond interactions with the ethylene glycol tail of the BTA-EG_6_ molecule (Figs. 5*B* and S10). The major difference between the top scoring poses for BTA-EG_6_ in site 1 involved small changes in the conformation of the oligo (ethylene glycol) chain of BTA-EG_6_, which was flexible and largely solvent exposed. For G2, the top poses in site 1 revealed the benzene ring having a hydrophobic interaction with I45 and an H-bond interaction with E492 (Figs. S9 and S11).

For site 2, the top scoring poses indicate that BTA-EG_6_ could, in principle, form hydrophobic interactions with residue I93, as well as cation–π and C–H–π interactions with residues R217 and E215, respectively (Figs. S9 and S12). Similarly, for G2, the top poses revealed possible hydrophobic interactions with I93 in binding site 2. These docking studies suggest that G2 could also form H-bond and cation–π interactions with E215 and R217 in site 2, respectively (Figs. 5*B* and S13).

We selected the four residues in site 1 that are proposed to be involved in the binding of BTA-EG_6_ and fascin-1—A137, I45, E492, and R389—as candidates for site-directed mutagenesis studies. Hydrophobic residues A137 and I45 are buried inside the cleft formed between β−trefoils 1 and 4 (Fig. 5*B*). Fascin-1 mutations A137K and I45Q were made to enhance binding to fascin-1 by potentially introducing H-bond interactions and cation–π interactions between BTA-EG_6_ and fascin-1 at site 1. As discussed, E492 is proposed to be involved in H-bond interactions with both BTA-EG_6_ and G2 at site 1, whereas residue R389 is proposed to be involved in H-bonding interactions with BTA-EG_6_ (Figs. 5*B* and S9–S11); fascin-1 mutants E492A and R389A were, therefore, designed to diminish these interactions and reduce binding. At site 2, we selected three residues—R217, E215, and I93—for site-directed mutagenesis studies. Hydrophobic residue I93 is buried inside the cleft between β-trefoils 1 and 2. The mutation I93W was selected to introduce a bulky group that we predicted could disrupt binding of either BTA-EG_6_ or G2 to site 2 on fascin-1 if they bound to that site. Residue R217 is predicted to potentially be involved in cation–π interactions with both BTA-EG_6_ and G2, whereas E215 is predicted to form C–H–π and H-bond interactions with BTA-EG_6_ and G2, respectively, at site 2 (Figs. 5*B*, S9, S12 and S13). Fascin-1 mutations R217A and E215A were, therefore, designed to disrupt these interactions and reduce the binding of either small molecule to fascin-1 if they bound to site 2.

To experimentally validate the hypothesis from the *in silico* molecular docking studies and to gain evidence of whether the BTA compounds could bind to site 1, site 2, or both, we generated, recombinantly expressed, and purified all seven fascin-1 mutant proteins—A137K, I45Q, R389A, E492A, R217A, E215A, and I93W. We confirmed by CD (Fig. S14) that their folding was similar to that of WT fascin-1. All seven of these fascin-1 mutant proteins also exhibited similar activity as WT fascin-1 for promoting bundling of actin filaments (Figs. 5*C* and S15). Next, we carried out binding studies by ITC to assess the binding affinity of site 1 fascin-1 mutant proteins to both BTA-EG_6_ and G2 (Figs. S16 and S17). As predicted from the *in silico* molecular docking studies, BTA-EG_6_ exhibited enhanced binding affinity to fascin-1 mutant proteins A137K and I45Q, whereas reduced binding affinity was observed to fascin-1 mutant proteins R389A and E492A compared with WT fascin-1 (Figs. 5*D* and S16). However, none of the site 1 mutations had any significant effect on the binding affinity of G2 to fascin-1 mutant proteins compared with WT fascin-1 (Figs. 5*E* and S17).

We then evaluated the binding affinity of BTA-EG_6_ and G2 to site 2 fascin-1 mutant proteins. We found that none of the site 2 mutations significantly changed the binding of BTA-EG_6_ to fascin-1 mutant proteins compared with WT fascin-1 (Figs. 5*D* and S18), whereas all the site 2 mutations diminished the binding of G2 to fascin-1 mutant proteins compared with WT fascin-1 (Figs. 5*E* and S19). Collectively, the binding data of the seven fascin-1 mutant proteins strongly suggest that BTA compounds are likely to bind to proposed binding site 1 on fascin-1, whereas G2 likely binds to proposed binding site 2, which could explain their opposing effects on fascin-1-mediated bundling of actin filaments (Fig. 3*B*). Additional experiments using solution NMR have also provided structural evidence for site 1 as the proposed location of the binding pocket of the BTA compounds in fascin-1 (data not shown).

## Discussion

Fascin-1 is an actin bundling protein and is well known for its role in the formation of filopodia in cells (22, 43). In healthy adults, high levels of fascin-1 are found in the brain as well as in mesenchymal cells (44). The dynamic nature of the actin cytoskeleton in neurons has led to extensive research into the role of fascin-1 and other actin binding proteins in neuronal tissue (45, 46). Fascin-1 is found to localize within filopodia found on the leading edge of axonal growth cones in developing neurons (47). Fascin-1 has also been reported to affect dendritic arborization in class III neurons in *Drosophila* (48), and fascin-1-pathway modulators have been found to affect *drosophila* neurite curvature (49). However, the direct role of fascin-1 in dendritic spine formation in mature mammalian neurons is poorly understood. We show that fascin-1 is present throughout the dendrites of mature neurons and can localize in dendritic protrusions consistent with dendritic spines (Fig. S4).

BTA-EG_4_, BTA-EG_6_, and other benzothiazole amphiphiles have been shown to increase spine density in mouse (*in vivo* and *in vitro*) (29), rat (*in vitro*) (16), and human induced pluripotent stem cell–derived neurons (50). In mice, they have also been shown to enhance memory and learning in both a 3× Tg AD model and in WT mice (21, 29, 30). The correlation between enhanced spine density and improved cognitive performance is supported by several foundational studies. In hippocampus-dependent associative learning tasks such as trace eyeblink conditioning, the dendritic spine density on CA1 pyramidal neurons increases significantly only in animals that learn the association; animals exposed to unpaired stimuli or treated with *N*-methyl-d-aspartate receptor antagonists fail to show this effect, despite identical experimental conditions (51). Additional studies confirm that learning-induced synaptic plasticity such as long-term potentiation is accompanied by structural remodeling of spines, including increases in spine number, volume, and complexity, supporting their role in memory encoding (52). Moreover, studies on the role of estrogen and stress in both male and female rats suggest that an increase in spine density creates an opportunity for efficient and rapid formation of new memories (53).

In the present study, photoaffinity-labeling studies using human brain cortex lysates showed that these BTA compounds target fascin-1 with high specificity (Fig. 1). Fascin-1 knockdown in primary neurons led to a decrease in spine density (Fig. 2, *D* and *E*) and negated the capability of BTA-EG_4_ and BTA-EG_6_ to promote spine density increases that were observed with endogenous levels of fascin-1. Both BTA-EG_4_ and BTA-EG_6_ were shown to have no significant effect on fascin-1 expression in primary neurons (Fig. S5), suggesting that these molecules exert their phenotypic activity of promoting increases in dendritic spine density by directly impacting the dynamic function of endogenous fascin-1 rather than by a mechanism that involves proteome-level changes in expression. This hypothesis is further supported by previous studies showing that the timescale of benzothiazole amphiphiles for promoting increases in spine density in primary neurons occurs within hours and exerts a maximal effect within 24 h, which favors a mechanism involving cytoskeletal reorganization rather than affecting changes in protein expression (16).

While there are a few previous reports of molecules that target fascin-1 (31, 32, 54, 55), all the molecules reported to date inhibit the actin-bundling activity of fascin-1. We found that both BTA-EG_4_ and BTA-EG_6_ can enhance the actin-bundling activity of fascin-1 (Fig. 3, *A* and *B*), which, to our knowledge, is unprecedented. G2, a known fascin-1 inhibitor (31, 32), was found to inhibit actin bundling mediated by fascin-1 (Fig. 3, *A* and *B*). TMT MS experiments with recombinantly expressed fascin-1 and human brain cortex lysates support that BTA compounds modulate, either directly or indirectly, the association of fascin-1 with actin and biological processes related to actin organization (Fig. 4), again supporting a mechanism where these compounds directly affect actin dynamics to exert their phenotypic activity in neurons.

Computational and mutagenesis studies provide some insight into how BTA-EG_4_ and BTA-EG_6_ could be mechanistically and functionally unique compared with other known fascin-1-binding molecules. *In silico* molecular docking studies revealed two major probable sites on fascin-1 for the binding of small molecules: site 1, between β-trefoils 1 and 4 (Fig. 5, *A* and *B*), is located near a purported actin-binding site on fascin-1 (38, 39), and site 2, between β-trefoils 1 and 2, has been reported to be the binding site for G2 analogs (31). We generated four mutants of fascin-1 within site 1 and found that all these mutations affected the binding affinity of BTA-EG_6_ to fascin-1 (Fig. 5*D*), but none of the site 1 mutations affected the binding affinity of G2 to fascin-1 (Fig. 5*E*), suggesting that BTA compounds, but not G2, can bind to site 1. We also generated three mutants of fascin-1 within site 2 and found that none of these mutations affected the binding affinity of BTA-EG_6_ to fascin-1 (Fig. 5*D*), but all the mutations affected the binding affinity of G2 to fascin-1 (Fig. 5*E*), suggesting that site 2 is a likely binding site for G2 but not for BTA compounds. While additional structural studies are under way to identify the exact binding mode of benzothiazole amphiphiles to site 1 on fascin-1, site 1 represents a previously unexplored binding pocket on fascin-1 that we show can be targeted by small molecules and lead to increased actin bundling and increased dendritic spine density in neurons.

In conclusion, we used a photoaffinity-labeling strategy to identify fascin-1 as the major cellular target of BTA-EG_4_ and BTA-EG_6_. We also showed that BTA compounds enhance the capability of fascin-1 to bundle actin filaments, increase the association of fascin-1 with actin and other actin-binding proteins in brain homogenates, and promote the formation of dendritic spines in neurons through targeting a newly uncovered binding site on fascin-1. This work presents a first step in providing a molecular mechanistic rationale for the capability of BTA compounds to improve memory and learning through promoting dendritic spine formation, which could lead to a novel strategy for treating NDDs and other memory impairment disorders.

## Experimental procedures

### Study approval

Use of APP/PS1 mouse brain tissue and rat primary neurons was in accordance with approved protocols from the UC San Diego Institutional Animal Care and Use Committee. The Guide for the Use and Care of Laboratory Animals published by the National Institutes of Health was followed, and protocols were completed in strict accordance with the practices described therein. Human brain tissue was obtained in accordance with UC San Diego IRB for human research and abides by the Declaration of Helsinki Principles. The Shiley–Marcos Alzheimer’s Disease Research Center at UC San Diego studied tissue donors both neurologically and psychometrically. Patient brains were collected by the UC San Diego Alzheimer’s Disease Research Center Neuropathology core upon autopsy.

### Culturing of SHSY-5Y and PC12 cells

SHSY-5Y cells were cultured in tissue culture–treated dishes (Genesee; catalog no.: 25-203) in modified Eagles’s medium:F12 (Gibco) media at a 1:1 ratio in a 37 °C humidified incubator as previously described (56). PC12 cells were maintained in a 37° humidified incubator with 5% CO_2_ in modified Eagles’s medium supplemented with 10% heat-inactivated horse serum (Gibco) and 5% fetal bovine serum (Gibco).

### Procedure for photoaffinity labeling of proteins in lysates

Brain tissue (1–2 g) or SHSY-5Y cells at 70 to 90% confluence from five 15 cm plates (Genesee; catalog no.: 25-203) harvested with typsin–EDTA 0.25% (Life Technologies; catalog no.: 25200056) were lysed by incubation in 5 ml hypotonic lysis buffer (20 mM Tris [pH 7.4]) for 30 min followed by lysis using a Dounce Homogenizer. Lysates were diluted to 1 mg/ml as assessed by bicinchoninic acid assay. Compound 1 and compound 2 were added to 1 ml lysates to a total concentration of 100 μM and 1% dimethyl sulfoxide (DMSO) (v/v). Samples were incubated for 30 min in the dark at 4 °C. Samples with UV treatment were transferred to a 96-well plate and exposed to long-wave UV light for 20 min at 4 °C. Proteins were separated by SDS-PAGE and transferred to a nitrocellulose membrane. Membranes were blocked for 1 h using 5% (W/V) blocking buffer (bovine serum albumin [BSA] in Tris-buffered saline solution with 0.1% Tween-20 [TBST]). Biotinylated molecules were detected using a streptavidin-conjugated HRP in 2% blocking buffer for 1 h followed by three washes in TBST and incubation with enhanced chemiluminescence reagent for 10 min and captured on film (Amersham Hyperfilm Genesee Scientific; catalog no.: 83-620).

### Affinity pulldowns of labeled targets

Photoaffinity-labeled proteins from SHSY-5Y cells were incubated with Neutravidin-agarose beads (Pierce; catalog no.: 29281), washed in lysis buffer (20 mM Tris, pH 7.4), then mixed with samples to afford a slurry (9:1 sample:neutravidin–agarose slurry), and shaken for 1 h at room temperature. The mixture was collected and centrifuged. The supernatant was removed, and wash buffer (20 mM Tris, pH 7.4, 2% SDS) was added. The beads were incubated in the wash buffer for 3 min with occasional vortexing. The slurry was centrifuged, and the supernatant was removed. This was performed a total of three times. The beads were suspended in 4XLDS sample buffer (Novex; catalog no.: NP0008) and were heated at 70 °C for 10 min to elute the proteins. Proteins were separated by SDS-PAGE.

### Sample preparation for LC–MS/MS analysis

Proteins from affinity pulldown and separated by SDS-PAGE were stained with a SilverQuest Silver Staining Kit (ThermoFisher; catalog no.: LC6070) according to the manufacturer’s instructions. Bands that appeared in the lane with treated sample but not in the lane with untreated sample were excised. The bands were destained, stored in MilliQ water, and submitted for tandem MS analysis.

### Sample sequencing using MS

The selected excised bands were reduced and alkylated with iodoacetamide, prior to trypsin digestion. The proteins were trypsinized for 45 min at 4 °C and incubated at 37 °C overnight. The supernatant containing the trypsin peptides was dried in a speed vac and stored at −20 °C for further analysis. Trypsin-digested samples were analyzed by LC–MS/MS using nanospray ionization on an Orbitrap fusion Lumos hybrid mass spectrometer (Thermo) interfaced with nanoscale reversed-phase UPLC (Thermo Dionex UltiMate 3000 RSLC nano system) using 25 cm, 75-micron ID glass capillary packed with 1.7-μm C18 BEH beads (Waters corporation) and separated on a linear gradient (5–80%) of ACN at a flow rate of 375 nl/min for 1 h in 0.1% formic acid. Mass spectrometer parameters used are (1) MS1 survey scan using the orbitrap detector (mass range [*m/z*]: 400 to 1500 [using quadrupole isolation], 60,000 resolution setting, spray voltage of 2200 V, ion transfer tube temperature of 285 °C, automatic gain control target of 400,000, and maximum injection time of 50 ms); (2) data-dependent scans (top speed for most intense ions, with charge state set to only include +2–5 ions, and an exclusion time of 5 s, while selecting ions with minimal intensities of 50,000 at which the collision event was carried out in the high-energy collision cell (higher-energy collisional dissociation collision energy of 30%), and the fragment masses were analyzed in the ion trap mass analyzer (with ion trap scan rate turbo, first mass *m/z* was 100, automatic gain control target of 5000, and maximum injection time of 35 ms). Protein identification and label-free quantification were carried out using Peaks Studio 8.5 (Bioinformatics solutions, Inc).

### Western blot confirmation of fascin-1 as a target of photoaffinity labeling

The proteins from affinity pulldown after SDS-PAGE separation were transferred to a nitrocellulose membrane, blocked for 1 h using 5% (w/v) blocking buffer (5% BSA in TBST), and probed for fascin-1 using a mouse ant–fascin-1 antibody (MAB3582) at 1:10,000 dilution in 2% blocking buffer overnight at 4 °C. This was followed by three washes in TBST at room temperature after which the membrane was labeled with goat anti-mouse HRP (Cytiva; catalog no.: NA931) in 2% blocking buffer for 1 h at room temperature followed by three washes in TBST at room temperature. The membrane was then incubated with enhanced chemiluminescence reagent for 10 min and imaged using Amersham A680 RGB imager.

### Dendritic spine analysis of primary neurons

Dissociated neurons from Sprague–Dawley 1-day-old (P1) male and female rat pups were prepared and maintained as previously described (57, 58, 59).

### Knockdown of fascin-1 in primary neurons

Cultured rat hippocampal neurons were transduced at 7DIV with 1 μl of AAV, either Fascin-shRNA-GFP-AAV (Vector Builder ID: VB230227-1603ujx) or Scramble-shRNA-GFP-AAV (Vector Builder ID: VB230505-1423kns), diluted in 1:20 Hank’s balanced salt solution. After 11 days, neurons were fixed and immunostained for fascin-1, GFP, and MAP2. For untreated samples, cells were fixed at 19DIV and immunostained with αGFP (Life Technologies; catalog no.: A11122) antibody. For treated cells, 5 μl of blinded sample was added to 1 ml of conditioned neuronal media 24 h prior to fixation.

### Knockdown of fascin-1 in PC12 cells

Cells were passaged 24 h prior to infection and plated to be around 70% confluent upon infection. AAV packaged viral particles were added at a multiplicity of infection of 10. Cells were harvested 3 days after infection using IP lysis buffer (Pierce) and following the manufacturer’s protocol and supplemented with cOmplete EDTA free protease (Roche; catalog no.: 11836170001) inhibitors following the manufacturer’s protocol. Lysates were normalized to 1 mg/ml total protein and separated *via* SDS-PAGE and transferred to a polyvinylidene difluoride membrane. Fluorescence intensities of fascin-1 normalized to GAPDH were analyzed using Image Studio Lite software (LI-COR).

### Dendritic spine density and morphology analysis

Neurons were analyzed in a blinded fashion using ImageJ (National Institutes of Health). The secondary dendritic shafts were straightened using an ImageJ straighten algorithm (60) and then cropped to 30 μm in length. The spines along the shaft were manually analyzed to count the number of spines and to measure the length and width of each spine. Data were collected in Microsoft Excel, and statistical analyses were performed using GraphPad Prism (GraphPad Software, Inc).

### Immunostaining of primary neurons

Primary neurons were fixed using 4% paraformaldehyde/sucrose solution in PBS solution with magnesium and calcium (PBS–MC) at room temperature for 10 min. Neurons were then permeabilized with 2% BSA and 0.25% Triton X-100 in PBS-MC at room temperature for 20 min, then blocked for 6 h at 4 °C in 5% BSA in PBS-MC. Primary antibodies for anti-fascin-1 (Millipore; catalog no.: MAB3582; 1:1000 dilution), anti-MAP2 (Abcam; catalog no.: 5392; 1:1000 or 1:5000 dilution), or anti-GFP (Life Technologies; catalog no.: A11122; 1:1000 dilution) were introduced in 2% BSA in PBS-MC and incubated overnight at 4 °C. Secondary antibodies were added 1:1000 for 1 h at room temperature, and 1× Hoechst stain (Sigma; catalog no.: B2261) was added for the last 10 min. Neurons were imaged with a Leica DMI6000 inverted microscope with the following specifications: A Yokogawa Nikon spinning disk confocal head. Orca ER high-resolution black and white cooled CCD camera (6.45 μm/pixel at 1×). Plan Apochromat 63×/1.4 numerical aperture objective. An argon/krypton air-cooled laser for 405 nm/140 mW, 100 mW/561 nm, and 140 mW/637 nm.

### GST-fascin-1 pulldowns and TMT analysis

Glutathione magnetic beads (Pierce; catalog no.: 78601) were washed three times in hypotonic lysis buffer (20 mM Tris [pH 8.0]). GST-fascin-1 or GST were loaded onto magnetic beads. Protein was added to the beads with 16 μl of 25% slurry glutathione magnetic beads and 20 μg GST-fascin-1 per trial. An equimolar amount of GST was prepared per control. The beads and protein were incubated while preparing the lysate (∼2 h) at 4 °C. After incubation, the beads were pulled to the side of the tube using a magnet and washed once with hypotonic lysis buffer immediately before being resuspended in an appropriate volume (∼25 μl per sample) before being added to the lysate.

Lysate was prepared by resuspending 1 g brain tissue in 3 to 5 ml cold hypotonic lysis buffer with protease inhibitor cocktail (Roche) and allowed to incubate for 30 min. Tissue was then homogenized by using a Dounce homogenizer. The tissue was ground extensively with first the loose and then the tight homogenizer rods on ice. Lysate was centrifuged at 18,000*g* for 20 min, and the soluble fraction was collected. Concentration was verified by bicinchoninic acid assay (Pierce; catalog no.: 23228) following the manufacturer’s protocol, and the lysate was diluted to 1 mg/ml total protein.

BTA-EG_4_, BTA-EG_6_, or DMSO (control) were added to the lysate to a final concentration of 100 μM compound and 0.1% DMSO. Preloaded beads were added to the lysate and incubated at 4 °C for 2 h. The beads were washed three times using cold wash buffer (0.1% Triton X-100 in PBS) using a magnet to pull the beads to the side of the tube prior to each wash. Samples were eluted by adding 25 μl of 8 M urea, 50 mM Hepes (pH 8.0) to the beads, agitating gently by flicking, then pulling the beads to the side with a magnet, and collecting the protein solution that was eluted off the beads. The elution step was repeated three times. Protein samples were then frozen at −80 °C until being sent for TMT MS. Trypsin digestion, TMT labeling and fractionation, and LC MS2/MS3 analysis were performed as previously described (61).

### Cloning procedure

Overhang PCR was used to create a fascin-1 amplicon containing an EcoRI restriction site, a thrombin cleavage sequence at the 5′ end, and a BamHI restriction site at the 3′ end. Oligonucleotides were synthesized by Eton Bioscience, Inc. PCR was performed using Phusion Flash High Fidelity PCR Master Mix (ThermoFisher; ccatalog no.: F548S). The PCR product was run on an agarose gel in TAE buffer containing 1X SYBR Safe DNA Gel Stain (Invitrogen). The band containing the amplicon was excised and purified from the gel using the Wizard SV Gel and PCR Clean-Up System (Promega; catalog no.: A9282). PCR product and the pGEX-5X-2 vector (GE Life Sciences) were separately digested using BamHI and EcoRI in 1X CutSmart buffer (New England BioLabs) for 1.5 h at 37 °C followed by a calf intestinal alkaline phosphatase treatment at 37 °C for 1 h (New England BioLabs). The DNA was purified, and the digested vector and insert were mixed in a 4:1 ratio by weight. DNA ligase (New England BioLabs) was added, and the ligation reaction was incubated for 10 min at room temperature. Subsequently, the mixture was heated at 65 °C for 10 min to inactivate the DNA ligase. The mixture was then transformed into TOP10 competent cells (ThermoFisher) according to the manufacturer’s instructions. After transformation, the TOP10 cells were plated on LB Amp-100 agar plates, and the plates were incubated at 37 °C overnight. The next day, six colonies were used to inoculate 12 ml LB Amp-100. The cultures were incubated overnight at 37 °C with shaking. The next day, the TOP10 cells were pelleted, and the plasmid was purified using a QIAprep Spin Miniprep Kit (Qiagen). The amount of purified DNA was quantified by gel, and sequencing confirmed the sequence of the insert.

### Expression and purification of WT fascin-1

DNA plasmid from TOP10 cells was purified using QIAprep Spin Miniprep Kit (Qiagen) and transformed into BL21-DE3 (New England BioLabs) cells following the manufacturer’s protocol. Individual clones were grown in 5 to 50 ml LB-Amp-100 overnight at 37 °C with shaking. The culture was then transferred to a 1 l flask containing 2XYT-Amp-100. Cultures were grown at 37 °C with shaking to an absorbance of ∼0.6 to 0.8 at 600 nm, followed by induction at 17 °C with 500 μg/ml IPTG (Apex). The cultures were left overnight, harvested by centrifugation, and frozen at −80 °C until purification.

Cells were lysed by sonicating on ice in resuspension buffer (20 mM Tris–HCl [pH 8.0], 150 mM NaCl). The lysate was centrifuged at 18,000*g* for 30 min to remove the cell debris. The supernatant was then incubated for at least 2 h with 5 ml of glutathione sepharose beads (Sigma; catalog no.: 17075605) per liter culture at 4 °C. After extensive washing with resuspension buffer, the beads were resuspended in thrombin cleavage buffer (20 mM Tris–HCl, pH 8.0, 150 mM NaCl, 2 mM CaCl_2_, and 1 mM DTT). Fascin-1 was released from the beads by incubation overnight with 40 to 100 U of thrombin at 4 °C per liter culture. After elution, 0.2 mM phenylmethylsulphonyl fluoride was added to inactivate the remnant thrombin activity. The fascin-1 protein was further concentrated with Amicon 30 molecular weight cutoff protein concentrator (Millipore) to greater than 10 mg/ml.

The purity of recombinant fascin-1 was determined by SDS-PAGE, and the identity was confirmed by Western blot using anti-fascin-1 antibody (MAB3582).

### Site-directed mutagenesis of fascin-1

Q5 Site-Directed Mutagenesis Kit (New England BioLabs) was used to introduce point mutations in fascin-1. Both forward and reverse primers for specific mutations were designed, and PCR was carried out using standard conditions as per the manufacturer’s specifications. The identity was confirmed by DNA sequencing.

### Expression and purification of fascin-1 mutants

The same protocol for expression and purification as that for WT fascin-1 was followed. Protein purity was assessed visually by SDS-PAGE, and protein identity was confirmed by Western blot using an anti-fascin-1 antibody (MAB3582).

### Circular dichroism

CD spectra of WT and fascin-1 mutant proteins were collected in the range of 195 to 300 nm using a Jasco J-810 spectropolarimeter. All samples were prepared in Tris buffer (2 mM Tris, pH 7.4, 1 mM DTT, 2 mM MgCl_2_), and the experiments were performed at 25 °C. The CD spectra were normalized with respect to protein concentration (0.5 Μm,) and the pathlength of the cuvette used (0.1 cm).

Molar ellipticity was calculated using the following formula and averaged across three scans:

[θ](molar ellipticity) = MW × 100 × θ/c × d (deg∙ cm^2^∙dmol^−1^) (c in mg/ml; d in cm)

[θ](mean residue ellipticity) = MRW × θ/10c × d (deg.cm^2^.dmol^−1^)

MRW (mean residue weight) = M/(N-1) (approximately 110 ± 5 Da)

### Slow speed actin sedimentation assay

F-actin bundling activity by fascin-1 was measured by a slow-speed centrifugation assay. Monomeric rabbit G-actin (Cytoskeleton, Inc) was induced to polymerize at room temperature in F-actin buffer (20 mM Tris–HCl, pH 8.0, 1 mM ATP, 1 mM DTT, 2 mM MgCl_2_, and 100 mM KCl) to a final concentration of 10 μM. Recombinant fascin-1 proteins (WT or mutant) were dissolved in polymerization buffer (2 mM Tris, 1 mM ATP, 1 mM DTT, 2 mM MgCl_2_, 100 mM KCl, pH 8.0) to a final concentration of 0.2 to 20 μM. BTA-EG_6_ or G2 (with 1% DMSO) (Xcess Biosciences) were both dissolved in polymerization buffer to a final concentration of 20 μM. Equal volumes of fascin-1 solution were subsequently incubated with F-actin for 60 min at room temperature and centrifuged for 30 min at 10,000*g*. Both supernatant and pellet were dissolved in an equivalent volume of SDS sample buffer (Novex) and then run on SDS-PAGE. The fraction of actin bundled was measured by quantifying the intensities of actin protein band in pellets compared with total actin.

### Isothermal titration calorimetry

Calorimetric measurements were carried out using a Low Volume ITC instrument (TA Instruments). The ligand (BTA-EG_6_ or G2) was titrated to the protein solution (fascin-1 or fascin-1 mutant proteins) present in the 350 μl sample cell. All measurements were carried out at 25 °C. The protein was dialyzed in 50 mM Hepes buffer (pH 7.5), 150 mM NaCl to a concentration of 100 μM. The ligand solution contained 1 mM of the small molecule (BTA-EG_6_ or G2) dissolved in the same buffer as the protein. Data were analyzed using the NanoAnalyze software (TA Instruments) for fitting points to the independent binding model. Experimental heats of the protein small-molecule titration were corrected for heats of dilution of the ligands into buffer alone.

### Computational docking studies

All steps for the docking experiments were carried out using Schrodinger suite 2019-20. Crystallographic structure of fascin-1 was retrieved from RCSB Protein Data Bank, Protein Data Bank ID: 1DFC (38). The protein was prepared using Maestro’s Protein Preparation Wizard (62). The SiteMap tool (41) was used to identify two ligand binding pockets on fascin-1. The receptor grids were then generated at the center of mass of the pocket residues and were made large enough to contain the binding site of the protein and the surrounding surface regions. Site 1 included residues 22, 27, 29, 39, 41, 68, 398, 408, 471, and 488; and site 2 included residues 11, 14, 16, 48, 49, 50, 58, 60, 62, 93, 95, 101, 103, 134, 213, 214, 215, 216, 217, 218, and 219. Ligands were constructed in Maestro (63), and LigPrep (62) was used to prepare the ligand for molecular docking. Each generated ligand was docked into both binding sites using Glide (64) with extra precision docking and default parameters to generate 100 poses for the ligand–receptor complex. The poses were clustered together using a cutoff RMSD value of 2 Å. The ligand interaction tool of the Molecular Operating Environment software (https://www.chemcomp.com/Products.htm) was used to study the expected interactions between ligand and protein residues in the binding sites. All the docked pose images were generated using Chimera (65), and 2D interactions maps were obtained from Molecular Operating Environment.

## Data availability

All data are available in the main article and the supporting information. Correspondence and requests should be directed to the jerryyang@ucsd.edu.

## Supporting information

This article contains supporting information (66, 67).

## Conflict of interest

J. Y. is a founder, equity interest holder, and advisor for Amydis, Inc. All other authors declare that they have no conflicts of interest with the contents of this article.
