# Supplementary material for: Nootropic benzothiazoles promote dendritic spine formation by targeting fascin-1
Source: J Biol Chem. 2025 Aug 8;301(9):110572. doi: 10.1016/j.jbc.2025.110572 (PMC12926044; doi:10.1016/j.jbc.2025.110572)
Supplement: Supplementary Materials [file mmc2.docx]

Supplementary Materials for

Nootropic Benzothiazoles Promote Dendritic Spine Formation by Targeting Fascin-1

Aashish Shivkumar^1,#^, Kyle R. Berg^1,#^, Kevin C. Sibucao^1,#^, Geoffray Leriche^1^, Lara E. Dozier^2^, Carla A. Espinoza^1^, Gentry N. Patrick^2^, Zied Gaieb^1^, Christian Seitz^1^, Rommie E. Amaro^1^, Hyun-Hee Park^3^, Hyang-Sook Hoe^3^, Jacob Wozniak^4^, David J. Gonzalez^4^, Saptarshi Sinha^5^, Pradipta Ghosh^5,6^, Jerry Yang^1,*^

Affiliations & Notes

1 Department of Chemistry and Biochemistry, University of California, San Diego, La Jolla CA 92093, USA

2 The Section of Neurobiology in the Division of Biological Sciences, University of California, San Diego, La Jolla, California 92093-0358, USA

3 Department of Neural Development and Disease, Korea Brain Research Institute (KBRI), 61 Cheomdan-ro, Dong-gu, Daegu, 41068, South Korea

4 Department of Pharmacology and the Skaggs School of Pharmacy and Pharmaceutical Sciences, University of California, San Diego, La Jolla, CA 92093, USA

5 Department of Cellular and Molecular Medicine, University of California San Diego, La Jolla, CA 92093, USA

6 Department of Medicine, University of California San Diego, La Jolla, CA 92093, USA

# These authors contributed equally

* Correspondence: [jerryyang@ucsd.edu](mailto:jerryyang@ucsd.edu)

**This file includes:**

Supplementary Note S1

Figures S1 to S19

Table S1

**Other supplementary materials for this manuscript include the following:**

Data S1

Supplementary Note S1

Synthetic Procedures

**Procedure A:** Synthesis of 5-((3aS,4S,6aR)-2-oxohexahydro-1H-thieno[3,4-d]imidazol-4-yl)-N-(2-(2-(2-(2-((4-(6-(3-(trifluoromethyl)-3H-diazirin-3-yl)benzo[d]thiazol-2-yl)phenyl)thio)ethoxy)ethoxy)ethoxy)ethyl)pentanamide (Compound **1**)

*Synthesis of the Functionalized Tetraethylene Glycol Tail*

**(1.1):** 2-(2-(2-(2-hydroxyethoxy)ethoxy)ethoxy)ethyl 4-methylbenzenesulfonate was synthesized by dissolving tetraethylene glycol (2.00g, 10.3mmol) in dichloromethane (DCM, 50mL) with stirring. Potassium Iodide (KI, 341 mg, 2.06mmol) and silver oxide (Ag_2_O, 3.58g, 15.4mmol) were added sequentially. Tosyl chloride (TsCl, 1.96g, 10.3mM) was added in portions. The reaction was stirred for 2 hours after complete addition of TsCl. The solids were removed by filtering over a pad of Celite. The resulting oil was concentrated under reduced pressure and purified via silica chromatography using Ethyl acetate (EtOAc) as the eluent to yield **1.1**, as a clear oil (1.96g, 55%). Characterization of **1.1**: ^1^H-NMR (400 MHz, CDCl_3_) δ 7.73 (d, 2H), 7.29 (d, 2H), 4.10 (t, 2H), 3.65-3.52 (m, 16H), 2.33 (s, 3H). ESI-MS (*m/z*) [M+Na]^+^ 371.1, [M+K]^+^ 387.1.

**(1.2):** 1-(2-(2-(2-(2-hydroxyethoxy)ethoxy)ethoxy)ethyl)triaza-1,2-dien-2-ium was synthesized by dissolving compound **1.1** (4.36g, 12.5mmol) in dry dimethylformamide (DMF, 15mL). Sodium azide (NaN_3_, 2.44g, 37.5mmol) was added to the reaction, which was then heated to 60°C and stirred overnight. After cooling to room temperature, a large excess of diethy ether (Et_2_O, 250mL) was added. The solids were removed by filtering over a pad of Celite. The resulting product was concentrated by evaporation under reduced pressure to yield **1.2** as a clear oil and used without further purification (2.79g, 98%). Characterization of **1.2**: ^1^H-NMR (400 MHz, CDCl_3_) δ 3.72-3.58 (m, 14H), 3.38 (t, 2H). ESI-MS (*m/z*) [M+NH_4_]^+^ 237.28, [M+Na]^+^ 242.04.

**(1.3):** 2-(2-(2-(2-aminoethoxy)ethoxy)ethoxy)ethan-1-ol was synthesized by dissolving compound **1.2** (2.50g, 11.4mmol) in 10mL methanol (MeOH). 625mg (approximately) palladium on carbon (Pd/C) was added and stirred at room temperature under an H_2_ atmosphere for 3 days. Filtering the crude over a pad of Celite, followed by removing solvent by reduced pressure yielded **1.3**, a clear oil and was used in the next step without further purification.

**(1.4):** Isopropyl (2-(2-(2-(2-hydroxyethoxy)ethoxy)ethoxy)ethyl)carbamate was synthesized by dissolving **1.3** (2.16g, 11.18mmol) in tetrahydrofuran (THF) and adding *tert*-butyl anhydride (Boc_2_O, 2.68g, 12.29mmol) with stirring for 4 hours. Solvent was removed by reduced pressure, and silica chromatography was used to purify the resulting oil using EtOAc as eluent to yield **1.4**, a clear oil (3.28g, 98% over two steps). Characterization of **1.4**: ^1^H-NMR (400 MHz, CDCl_3_) δ 3.73-3.59 (m, 14H), 3.32 (q, 2H), 1.43 (s, 9H). ESI-MS (*m/z*) [M+H]^+^ 294.92, [M+Na]^+^ 316.10.

**(1.5):** 2-methyl-4-oxo-3,8,11,14-tetraoxa-5-azahexadecan-16-yl 4-methylbenzenesulfonate was synthesized by dissolving **1.4** (1.50g, 5.11mmol) in DCM (15mL) and adding triethyl amine (Et_3_N, 1.42mL, 10.22mmol). The mixture was cooled to 0°C, upon which TsCl (1.95g, 10.22mmol) was added and stirred overnight. A solid precipitate was formed, and solvent was evaporated under reduced pressure. Silica chromatography was used to purify the product using a gradient from 1:1 hexanes/EtOAc to 1:3 hexanes/EtOAc as eluent to yield **1.5** as an oil (2.15g, 94%). Characterization of **1.5**: ^1^H-NMR (500 MHz, CDCl_3_) δ 7.76 (d, 2H), 7.31 (d, 2H), 4.99 (s, 1H), 4.13 (t, 2H), 3.66 (t, 2H), 3.58-3.55 (m, 8H), 3.49 (t, 2 H), 3.27 (m, 2H), 2.41 (s, 3H), 1.40 (s, 9H). ESI-MS (*m/z*) [M+H]^+^ 447.85, [M+Na]^+^ 470.06.

**(1.6):** isopropyl (2-(2-(2-(2-iodoethoxy)ethoxy)ethoxy)ethyl)carbamate was synthesized by dissolving **1.5** (1.00g, 2.23mmol) in dry acetone and adding sodium iodide (NaI, 1.34g, 8.94mmol). The reaction was heated to reflux and stirred overnight. Solids were removed by filtering over a pad of Celite and the flow-through was concentrated by reduced pressure and dissolved in EtOAc (30mL). The resulting crude was washed with 10% Na_2_S_2_O_3_ (20mL) and brine (20mL). The organic layer was extracted and dried over Na_2_SO_4_, then filtered to remove the Na_2_SO_4_. The solvent was evaporated under reduced pressure and yielded **1.6** as a clear oil (719mg, 80%). Characterization of **1.6**: ^1^H-NMR (500 MHz, CDCl3) δ 3.76 (t, 2H), 3.67-3.61 (m, 8H), 3.26 (t, 2H), 1.44 (s, 9H). ESI-MS (*m/z*) [M+H]^+^ 403.87, [M+Na]^+^ 426.05.

*Synthesis of the 2-Arylbenzothiazole Core*

**(1.7):** (4-iodophenyl)(methyl)sulfane was synthesized by cooling a solution of 4-bromothioanisole (1.50g, 7.39mmol) in THF (30mL) to -78°C in a dry ice-acetone bath. *Tert*-butyllithium (9.56mL, 1.7M solution in pentane, 16.26 mmol) was added dropwise to the mixture. The solution remained yellow after the addition of *tert*-butyllithium. I_2_ (2.06g, 8.13mmol) was first dissolved in THF, and then added to the solution dropwise. The solution became dark red after the addition of I_2_ and the mixture was returned to room temperature. A saturated solution of Na_2_S_2_O_3_ was added until the mixture became colorless. An organic layer was created by the addition of Et_2_O (50mL) and the layers were separated. The aqueous layer was again extracted with Et_2_O (2x50mL), and the organic layers were combined and dried over Na_2_SO_4_. Solvent was removed under reduced vacuum, and the compound **1.7** became green. Upon exposure to air, it became yellow (1.40g, 76%). Characterization of **1.7**: ^1^H NMR (CDCl_3_, 400 MHz) δ 7.56 (d, 2H), 6.97 (d, 2H), 2.44 (s, 3H) which is consistent with previous report^68^.

**(1.8):** N-methoxy-N-methylbenzo[d]thiazole-6-carboxamide was synthesized by first suspending benzothiazole-6-carboxylic acid (1.50g, 8.37mmol) in dry DCM (40mL). The solution clarified upon addition of diisopropylethylamine (DIPEA, 5.84mL, 33.48mmol). Hexafluorophosphate Azabenzotriazole Tetramethyl Uronium (HATU, 3.82g, 10.04mmol) was added, after which the reaction was stirred for 10 minutes. Next, *N,O*-dimethylhydroxylamine (950mg, 10.04mmol) was added, and the mixture was stirred for 1 hour. DCM was used to dilute the reaction mixture, and the organic layer was washed with 1M HCl (20mL) and saturated bicarbonate (20mL). The organic layer was collected and dried over Na_2_SO_4_, and the solvent removed under reduced pressure. Silica chromatography was used to purify the crude, using 1:3 Hexanes/EtOAc as eluent to yield product **1.8.** Characterization of **1.8**: ^1^H NMR (CDCl_3_, 500 MHz) δ 9.10 (s, 1H), 8.35 (s, 1H), 8.13 (d, 1H), 7.84 (dd, 1H), 3.53 (s, 3H), 3.39 (s, 3H). ESI-MS (*m/z*) [M+H]^+^ 223.16.

**(1.9):** N-methoxy-N-methyl-2-(4-(methylthio)phenyl)benzo[d]thiazole-6-carboxamide was synthesized by grinding together with a pestle and mortar **1.7** (1.35g, 5.40mmol), **1.8** (1.00g, 4.50mmol), PdCl_2_ (dppf) (3.29mg, 0.45mmol), PPh_3_ (236mg, 0.90mmol), and Ag_2_CO_3_ (2.48g, 9.00mmol). The mixture was placed in a screw cap tube. The mixture was suspended in water (5mL) and heated at 60°C overnight. Over the course of the reaction the mixture became black. DCM (5mL) was added, and the reaction mixture was filtered over a pad of Celite. The pad was washed with DCM (150mL), and the flow through was washed with water (50mL). The organic layer was separated and dried over Na_2_SO_4_. Silica chromatography was used to purify the crude using a 3:1 to 2:1 to 1:1 hexanes/EtOAc gradient as eluent to yield a white solid **1.9**. (891mg, 58%) Characterization of **1.9**: ^1^H NMR (CDCl_3_, 500 MHz) δ 8.26 (s, 1H), 8.03-7.98 (m, 3H), 7.81 (dd, 1H), 7.31 (d, 2H), 3.56 (s, 3H), 3.40 (s, 3H), 2.53 (s, 3H). ^13^C NMR (CDCl_3_, 500 MHz) δ 170.12, 169.28, 155.67, 143.68, 134.68, 130.70, 129.89, 128.08, 126.93, 126.06, 122.54, 122.51, 61.41, 34.02, 15.28. ESI-MS (*m/z*) [M+H]^+^ 345.20.

**(1.10):** 2-(4-(methylthio)phenyl)benzo[d]thiazole-6-carbaldehyde was synthesized by dissolving **1.9** (891mg, 2.59mmol) in dry THF (20mL) and cooling to 0°C. Lithium aluminum hydride (LAH, 2.84mL, 1M in THF, 2.84mmol) was added dropwise and stirred for 1 hour. EtOAc (20mL) was added to quench any remaining LAH. Next a solution of saturated Rochelle salt (30mL) was added, and the resulting biphasic mixture was vigorously stirred for 1 hour. The organic layer was removed, and DCM (2x20mL) was used to extract the aqueous layer. The organic layers were combined and dried over Na_2_SO_4_. The solvent was removed by reduced pressure, and the resulting solid crude was recrystallized from isopropanol (iPrOH) to yield **1.10** as an off-white solid (596mg, 80%). Characterization of **1.10**: ^1^H NMR (CDCl_3_, 500 MHz) δ 10.09 (s, 1H), 8.42 (s, 1H) 8.12 (d, 1H), 8.01 (d, 2H), 7.98 (dd, 1H), 7.32 (d, 2H), 2.54 (s, 3H). ^13^C NMR (CDCl_3_, 500 MHz) δ 191.35, 172.33, 158.34, 144.46, 135.63, 133.32, 129.57, 128.24, 127.69, 126.01, 124.51, 123.61, 15.22. ESI-MS (*m/z*) [M+H]^+^ 286.26, [M+MeOH+H]^+^ 318.30.

*Coupling the Functionalized Tetraethylene Glycol Tail with the 2-Arylbenzothiazole Core*

**(1.11):** 2-(4-(methylsulfinyl)phenyl)benzo[d]thiazole-6-carbaldehyde was synthesized by suspending **1.10** (260mg, 0.91mmol) and NaHCO_3_(306mg, 3.64mmol) in DCM (5mL) and cooled to 0°C. *meta*-chloroperbenzoic acid (mCPBA, 204mg, 0.911mmol, ~77% purity) was dissolved in DCM (5mL) and added dropwise. The reaction was stirred for 1 hour while remaining at 0°C. The crude was next diluted with DCM (20mL) and washed with 1M NaOH (10mL). Afterward the organic layer was collected and dried over Na_2_SO_4_. Reduced pressure was used to remove the solvent and silica chromatography was used to purify the crude using a gradient of DCM to 2% MeOH in DCM as eluent to yield **1.11** as a white solid (258mg, 94%). Characterization of **1.11**: ^1^H NMR (CDCl_3_, 500 MHz) δ 10.12 (s, 1H), 8.46 (d, 1 H), 8.28 (d, 2H), 8.19 (d, 1H), 8.03 (dd, 1H), 7.79 (d, 2H), 2.79 (s, 3H). ^13^C NMR (CDCl_3_, 500 MHz) 191.24, 170.88, 158.04, 149.66, 135.91, 135.67, 133.83, 128.83, 127.76, 124.74, 124.57, 124.26, 44.16. ESI-MS (*m/z*) [M+H]^+^ 302.25, [M+MeOH+H]^+^ 334.19.

**(1.12):** tert-butyl(2-(2-(2-(2-((4-(6-formylbenzo[d]thiazol-2-yl)phenyl)thio)ethoxy)ethoxy)ethoxy)ethyl) carbamate was synthesized by suspending **1.11** (160mg, 0.531mmol) in DCM (~2mL), and adding trifluoroacetic anhydride (TFAA, ~2mL). The reaction was refluxed for 1 hour and reduced pressure was used to remove the solvents. The crude was used in the next step and was dissolved in a solution of 50% Et_3_N in MeOH (~4 mL). The crude turned a red color and was stirred for 10 minutes. Reduced pressure was used to remove the solvents. Next, the crude was dissolved in dry THF (3mL) and K_2_CO_3_ (147mg, 1.06mmol) was added. **1.6** (107mg, 0.266mmol) was dissolved in THF and added to the mixture and stirred overnight. Reduced pressure was used to remove the solvent, and the crude was dissolved in EtOAc. The organic layer was removed and dried over Na_2_SO_4._ Silica Chromatography was used to purify the crude using a gradient of 2:1 Hexanes/EtOAc to 1:2 Hexanes/EtOAc as eluent to produce **1.12** as an off-white solid (87.3mg, 35%). Characterization of **1.12**: ^1^H NMR (CDCl_3_, 500 MHz) δ 10.11 (s, 1H), 8.44 (s, 1H), 8.15 (d, 1H), 8.03-8.00 (m, 3H), 7.43 (d, 2H), 3.76 (t, 2H), 3.66-3.62 (m, 8H), 3.54 (t, 2H), 3.32 (q, 2H), 3.25 (t, 2H), 1.43 (s, 9H). ^13^C NMR (CDCl_3_, 500 MHz) δ 191.36, 172.13, 158.31, 156.20, 142.34, 135.68, 133.40, 130.33, 128.34, 127.93, 127.69, 124.56, 123.71, 70.81, 70.77, 70.45, 69.86, 40.54, 32.10, 28.63. ESI-MS (*m/z*) [M+H]^+^ 547.14, [M+Na]^+^ 569.22.

*Synthesis of the Trifluoromethyl Diazirine*

**(1.13):** tert-butyl (2-(2-(2-(2-((4-(6-(2,2,2-trifluoro-1-hydroxyethyl)benzo[d]thiazol-2-yl)phenyl)thio)ethoxy)ethoxy)ethoxy)ethyl)carbamate was synthesized by dissolving **1.12** (51mg, 0.093mmol) in dry DMF (~2mL). A few grains (trace amount) of K_2_CO_3_ were added to the mixture. Trifluoromethyltrimethylsilane (TMSCF_3_) (~20µL) was added. The reaction was allowed to proceed for 30 minutes and monitored by TLC. When the reaction was complete, tetrabutylammoniumfluoride (TBAF) (~18 µL) was added to remove the TMS group. Silica chromatography was used to purify the crude using a gradient of 1:1 Hexanes/EtOAc as eluent to yield **1.13** as a white solid (17.2mg, 30%). Characterization of **1.13**: ^1^H NMR (CDCl_3_, 400 MHz) δ 8.05-8.03 (m, 2H), 7.96 (d, 2H), 7.57 (d, 1H), 7.39 (d, 2H), 5.20-5.15 (m, 1H), 3.74 (t, 2H), 3.66-3.60 (m, 8H), 3.50 (t, 2H), 3.28 (q, 2H), 3.21 (t, 2H), 1.43 (s, 9H). ESI-MS (*m/z*) [M+H]^+^ 617.14, [M+Na]^+^ 639.20.

**(1.14):** tert-butyl (2-(2-(2-(2-((4-(6-(2,2,2-trifluoroacetyl)benzo[d]thiazol-2-yl)phenyl)thio)ethoxy)ethoxy)ethoxy)ethyl)carbamate was synthesized by dissolving **1.13** (11mg, 0.018mmol) in dry DCM and cooling to 0°C in an ice bath. Next, Dess-Martin periodinane (DMP, 30mg, 0.071mmol) was added and the mixture was allowed to equilibrate to room temperature. The mixture was stirred for two hours. Additional DCM and saturated NaHCO_3_ were added, and the mixture was stirred until the organic layer clarified. The organic layer was removed. DCM (10mL) was used to extract the aqueous layer. Silica chromatography was used to purify the crude using a gradient of 3:1 Hexanes/EtOAc as eluent to produce the white solid **1.14** (5mg, 42%). Characterization of **1.14**: ^1^H NMR (CDCl_3_, 500 MHz) δ 8.65 (s, 1H), 8.20-8.02 (m, 2H), 8.03 (d, 2H), 7.43 (d, 2H), 3.76 (t, 2H), 3.69-3.61 (m, 8H), 3.54 (t, 2H), 3.32 (q, 2H), 3.25 (t, 2H), 1.43 (s, 9H). ESI-MS (*m/z*) [M+Na+H_2_O]^+^ 655.26, [M+Na+MeOH]^+^ 669.26.

**(1.15):** tert-butyl (E)-(2-(2-(2-(2-((4-(6-(2,2,2-trifluoro-1-(hydroxyimino)ethyl)benzo[d]thiazol-2-yl)phenyl)thio)ethoxy)ethoxy)ethoxy)ethyl)carbamate was synthesized by first dissolving **1.14** (16mg, 0.027mmol) in pyridine (2mL). NH_2_OH∙HCl (6mg, 0.08mmol) was added to the mixture and it was heated to 80°C with stirring for 6 hours. Solvent was removed by reduced pressure and silica chromatography was used to purify the crude using 1:2 Hexanes/EtOAc as eluent to produce **1.15** as a white solid (15.4mg, 94%)**.** Characterization of **1.15**: ^1^H NMR (CDCl_3_, 500 MHz) δ 8.11-8.07 (m, 1H), 8.04-8.00 (m, 1H), 7.94-7.90 (m, 2H), 7.62 (t, 1H), 7.35 (t, 2H), 3.73 (t, 2H), 3.67-3.65 (m, 8H), 3.55 (t, 2H), 3.33 (q, 2H), 3.20 (t, 2H), 1.41 (s, 9H). ESI-MS (*m/z*) [M+H]^+^ 630.08, [M+Na]^+^ 652.21.

**(1.16):** tert-butyl (2-(2-(2-(2-((4-(6-(3-(trifluoromethyl)diaziridin-3-yl)benzo[d]thiazol-2-yl)phenyl)thio)ethoxy)ethoxy)ethoxy)ethyl)carbamate was synthesized by dissolving **1.15** (15.4mg, 0.025mmol) in DCM (~2mL). Et_3_N (~7µL, 0.05mmol) was added. The solution was then cooled to 0°C. TsCl (9.5mg, 0.05mmol) was added, and the reaction was stirred for 1 hour. Reduced pressure was used to remove the solvent, and the crude was used in the next step without further purification. The crude was dissolved in Et_2_O (2mL), and placed in a thick-walled screw-cap tube and cooled to -78°C. After cooling, a cannula was used to transfer liquid ammonia (~2mL) to the solution. The tube was sealed and vigorously stirred, then warmed to room temperature. The reaction was stirred for an additional 4 hours. The tube was carefully opened, and the excess ammonia was removed by evaporation. Silica chromatography was used to purify the crude using 1:2 Hexanes/EtOAc as eluent to yield **1.16** as a white solid (10mg, 92%). Characterization of **1.16**: ^1^H NMR (500 MHz, CDCl_3_) δ 8.14 – 8.00 (m, 2H), 7.95 (t, *J* = 8.7 Hz, 2H), 7.65 – 7.57 (m, 1H), 7.43 – 7.33 (m, 2H), 5.09 (br s, 1H), 3.74 (t, *J* = 6.8 Hz, 2H), 3.65 (m, 8H), 3.54 (t, *J* = 5.2 Hz, 2H), 3.33 (q, *J* = 5.5 Hz, 2H), 3.21 (t, *J* = 6.8 Hz, 2H), 1.43 (s, 9H). ESI-MS (*m/z*) [M+H]^+^ 630.8, [M+Na]^+^ 652.21.

**(1.17):** tert-butyl (2-(2-(2-(2-((4-(6-(3-(trifluoromethyl)-3H-diazirin-3-yl)benzo[d]thiazol-2-yl)phenyl)thio)ethoxy)ethoxy)ethoxy)ethyl)carbamate was synthesized by dissolving **1.16** (10mg, 0.016mmol) in dry DCM (~2mL). MnO_2_ (~14mg, 0.16mmol), and the resulting suspension was stirred for 1 hour. Solids were removed by filtering over a pad of Celite. Silica chromatography was used to purify the crude using 1:1 Hexanes/EtOAc as eluent to produce the white solid **1.17** (7mg, 70%)**.** Characterization of **1.17**: ^1^H-NMR (400 MHz, CDCl_3_) δ 8.04 (d, 1H), 7.98 (d, 2H), 7.75 (s, 1H), 7.42 (d, 2H), 7.32 (d, 1H), 3.74 (t, 2H), 3.67-3.60 (m, 8H), 3.53 (t, 2H), 3.32 (q, 2H), 3.23 (t, 2H), 1.43 (s, 9H). ESI-MS (*m/z*) [M-N_2_+Na]^+^ 621.20, [M+Na]^+^ 649.10.

Synthesis of Compound **1**

**(1.18):** 2,5-dioxopyrrolidin-1-yl 5-((3aS,4S,6aR)-2-oxohexahydro-1H-thieno[3,4-d]imidazol-4-yl)pentanoate was synthesized by suspending Biotin (1.00 g, 4.09mmol) and N-hydrosuccinimide (471mg, 4.09mmol) in DMF. The suspension was heated until it clarified. Dicyclohexylcarbodiimide (DCC, 928mg, 4.50mmol) was added, followed by cooling the mixture to room temperature. The reaction was allowed to proceed overnight. The precipitate was filtered, and the solvent removed by reduced vacuum. Et_2_O was used to suspend the crude, which was then filtered. The remaining solids were recrystallized from iPrOH to produce a flakey white solid (970mg, 69%). Characterization of **1.18**: ^1^H-NMR (400 MHz, DMSO) δ 6.44 (s, 1H), 6.38 (s, 1H), 4.31 (t, 1H), 4.14 (t, 1H), 3.10 (m, 1H), 2.85-2.81 (m, 5H), 2.67 (t, 2H), 2.58 (d, 1), 1.68-1.61 (m, 3H), 1.53-1.40 (m, 3H). which is consistent with the previous report^69^.

**(**Compound **1):** 5-((3aS,4S,6aR)-2-oxohexahydro-1H-thieno[3,4-d]imidazol-4-yl)-N-(2-(2-(2-(2-((4-(6-(3-(trifluoromethyl)-3H-diazirin-3-yl)benzo[d]thiazol-2-yl)phenyl)thio)ethoxy)ethoxy)ethoxy)ethyl)pentanamide was synthesized by dissolving **1.17** (2.4mg, 0.0038mmol) in DCM (1mL). The mixture was cooled to 0°C. Trifluoroacetic acid (TFA, 1mL) was added dropwise. The solution turned a bright yellow color upon addition of TFA. The mixture was warmed to room temperature and stirred for 2 hours. Reduced vacuum was used to remove the solvent, and the crude was carried over to the next step without further purification. The crude was dissolved in DMF (1mL). DIPEA (3µL, 0.015 mmol) and **1.18** (2mg, 0.0057mmol) were added sequentially. The mixture was stirred for 4 hours, and reduced pressure was used to remove solvent. The crude was dissolved in DCM (6mL) and washed with 1M NaOH (4mL). DCM (6mL) was used to extract the aqueous layer. The combined organic layers were dried with Na_2_SO_4_. Silica chromatography was used to purify the crude using a gradient of DCM to 10% MeOH in DCM as eluent to yield Compound **1,** a white slightly tacky solid (1.8mg, 63%) Characterization of Compound **1**: ^1^H NMR (CDCl_3_, 500 MHz) δ 8.01 (d, 1H), 7.96 (d, 2H), 7.73 (s, 1H), 7.39 (d, 2H), 7.29 (d, 1H), 6.65 (s, 1H), 6.31 (s, 1H), 5.31 (s, 1H), 4.46 (t, 1H), 4.27 (t, 1H), 3.71 (t, 2H), 3.63-3.60 (m, 8H), 3.54 (t, 2H), 3.45-3.37 (m, 2H), 3.20 (t, 2H), 3.10-3.06 (m, 1H), 2.86 (dd, 1H), 2.70 (d, 1H), 2.22-2.15 (m, 2H), 1.72-1.58 (m, 4H), 1.41-1.37 (m, 2H). ESI-MS (*m/z*) [M-N_2_+Na]^+^ 747.37, [M+H]^+^ 753.22, [M+Na]^+^ 775.19. HR-MS (m/z) calculated for [M+Na]^+^ 775.1988; found [M+Na]^+^ 775.1986.

**Procedure B:** Synthesis of N-(2-(2-(2-(2-((4-(benzo[d]thiazol-2-yl)phenyl)thio)ethoxy)ethoxy)ethoxy)ethyl)-5-((3aS,4S,6aR)-2-oxohexahydro-1H-thieno[3,4-d]imidazol-4-yl)pentanamide (Compound **2**)

**(2.1):** 2-(4-(methylthio)phenyl)benzo[d]thiazole was synthesized by combining Benzothiazole (0.5g, 3.70mmol), compound **1.7** (1.1g, 4.44mmol), PdCl_2_(dppf) (270mg, 0.370mmol), PPh_3_ (194mg, 0.739mmol), and Ag_2_CO_3_ (2.04g, 7.39 mmol) with a pestle and mortar. The mixture was transferred to a screw cap tube. The solid mixture was suspended in water (5mL), heated to 60°C and stirred overnight. Over the course of the reaction the mixture turned black. DCM (10mL) was added to the reaction, and a pad of Celite was used to filter the solids. DCM (150mL) was used to wash the pad of Celite. Water (50mL) was used to wash the flowthrough. The organic layer was separated and dried with Na_2_SO_4_. Silica chromatography was used to purify the crude using a 9:1 to 4:1 hexanes/EtOAc gradient as eluent. The crude was then recrystallized from iPrOH to produce **2.1** as a white solid (506 mg, 53%)**.** Characterization of **2.1**: ^1^H NMR (CDCl_3_, 500 MHz) δ 8.05 (s, 1H), 8.01 (d, 2H), 7.90 (s, 1H), 7.49 (t, 1H), 7.38 (t, 1H), 7.33 (d, 2H), 2.55 (s, 1H). ^13^C NMR (CDCl_3_, 500 MHz) δ 167.81, 154.32, 142.97, 135.04, 130.25, 127.96, 126.54, 126.11, 125.29, 123.21, 121.79, 15.36. ESI-MS (*m/z*) [M+H]^+^ 258.32.

**(2.2):** 2-(4-(methylsulfinyl)phenyl)benzo[d]thiazole was synthesized by suspending **2.1**( 75mg, 0.29mmol) and NaHCO_3_ (98mg, 1.17mmol) in DCM (2mL), and then cooling the mixture to 0°C. mCPBA (65.4 mg, 0.29 mmol, ~77% purity) was dissolved in DCM (2mL) and added dropwise to the solution. At 0°C, the reaction was stirred for 2 hours. The crude was then diluted with DCM (15mL) and 1M NaOH (10mL) was added to wash the solution. The organic layer was removed and dried with Na_2_SO_4_. Reduced pressure was used to remove the solvent, and silica chromatography was used to purify the crude using EtOAc followed by a gradient of DCM to 2% MeOH in DCM as eluent to produce product **2.2** as a white solid (76mg, 95%)**.** Characterization of **2.2**: ^1^H NMR (CDCl_3_, 500 MHz) δ 8.26 (d, 2H), 8.11 (d, 1H), 7.95 (d, 1H), 7.78 (d, 1H), 7.53 (t, 1H), 7.44 (t, 1H), 2.79 (s, 3H). ^13^C NMR (CDCl_3_, 500 MHz) 166.46, 154.23, 148.58, 136.33, 135.34, 128.55, 126.88, 125.97, 124.42, 123.75, 121.99, 44.19. ESI-MS (*m/z*) [M+H]^+^ 274.15.

**(2.3):** tert-butyl (2-(2-(2-(2-((4-(benzo[d]thiazol-2-yl)phenyl)thio)ethoxy)ethoxy)ethoxy)ethyl)carbamate was synthesized by suspending **2.2** (20mg, 0.073mmol) in DCM (~2mL), and TFAA (~2mL was added). The reaction was refluxed for 1.5 hours, and reduced pressure used to reduce the solvents. The crude was carried over to the next step without further purification. 50% Et_3_N in MeOH (~4 mL) was used to dissolve the crude. The crude turned a red color and was stirred for 10 minutes. Reduced pressure was used to remove the solvents. The crude was then dissolved in dry THF (3mL), and K_2_CO_3_ (20mg, 0.148mmol) was added. Compound **1.6** (14.8mg, 0.037mmol) was dissolved in THF and added to the reaction. The reaction was allowed to proceed with stirring overnight. Reduced pressure was used to remove the solvents and the crude was dissolved in EtOAc. 1M NaOH (~10mL), water (10mL), and brine (10mL) were sequentially used to wash the organic layer, which was then dried over Na_2_SO_4_. Silica chromatography was used to purify the crude using a gradient of 1:1 Hexanes/EtOAc to produce **2.3** as an off-white solid (16.3mg, 85%). Characterization of **2.3**: ^1^H NMR (CDCl_3_, 500 MHz) δ 8.05 (d, 1H), 8.00 (d, 2H), 7.90 (d, 1H), 7.49 (t, 1H), 7.43-7.37 (m, 3H), 3.74 (t, 2H), 3.66-3.60 (m, 9H), 3.53 (t, 2H), 3.32 (q, 2H), 3.22 (t, 2H), 1.43 (s, 9H). ^13^C NMR (CDCl_3_, 500 MHz) δ 167.60, 156.21, 154.31, 140.71, 135.09, 131.09, 128.27, 128.07, 126.59, 125.39, 123.29, 121.83, 79.40, 76.66, 70.79, 70.72, 70.43, 69.93, 40.53, 32.30, 28.63. ESI-MS (*m/z*) [M+H]^+^ 519.07, [M+Na]^+^ 541.13.

**(**Compound **2):** N-(2-(2-(2-(2-((4-(benzo[d]thiazol-2-yl)phenyl)thio)ethoxy)ethoxy)ethoxy)ethyl)-5-((3aS,4S,6aR)-2-oxohexahydro-1H-thieno[3,4-d]imidazol-4-yl)pentanamide was synthesized by first dissolving compound **2.3** (16.3mg, 0.0314mmol) in DCM (2mL). The solution was cooled to 0°C, and TFA (2 mL) was added dropwise to the solution. The solution became bright yellow. The mixture was warmed to room temperature and stirred for 1 hour. Reduced vacuum was used to remove solvent, and the crude was used directly in the next step. The crude was dissolved in DMF (2mL). DIPEA was next added, and then compound **1.18** (12.8mg, 0.0377mmol). The reaction was stirred for 5 hours, and the solvent was removed by reduced pressure. DCM (6mL) was used to dissolve the crude, which was then washed with 1M NaOH (4mL). DCM (6mL) was then used to extract the aqueous layer. The organic layers were combined and then dried over Na_2_SO_4_. Silica chromatography was used to purify the crude using 8% MeOH in DCM as eluent to yield Compound **2** as a white slightly tacky solid (15.8mg, 78%). Characterization of Compound **2**: ^1^H NMR (CDCl_3_, 500 MHz) δ 8.04 (d, 1H), 7.99 (d, 2H), 7.89 (d, 1H), 7.48 (t, 1H), 7.42-7.36 (m, 3H), 6.84 (s, 1H), 6.70 (s, 1H), 5.66 (s, 1H), 4.46 (t, 1H), 4.26 (t, 1H), 3.72 (t, 2H), 3.65-3.59 (m, 8H), 3.55 (t, 2H), 3.47-3.36 (m, 2H), 3.21 (t, 2H), 3.10-3.06 (m, 1H), 2.87-2.83 (dd, 1H), 2.71 (d, 1H), 2.19 (t, 2H), 1.75-1.59 (m, 4H), 1.42-1.37 (m, 2H). ^13^C NMR (CDCl_3_, 500 MHz) δ 173.56, 167.54, 164.28, 154.24, 140.68, 135.04, 131.05, 128.23, 128.06, 126.60, 125.41, 123.24, 121.84, 70.64, 70.59, 70.23, 70.15, 69.84, 61.87, 60.36, 55.81, 40.72, 39.31, 36.18, 32.37, 29.89, 28.39, 28.26, 25.82. ESI-MS (*m/z*) [M+H]^+^ 645.38, [M+Na]^+^ 667.40. HR-MS (m/z) calculated for [M+Na]^+^ 667.2053; found [M+Na]^+^ 667.2052.


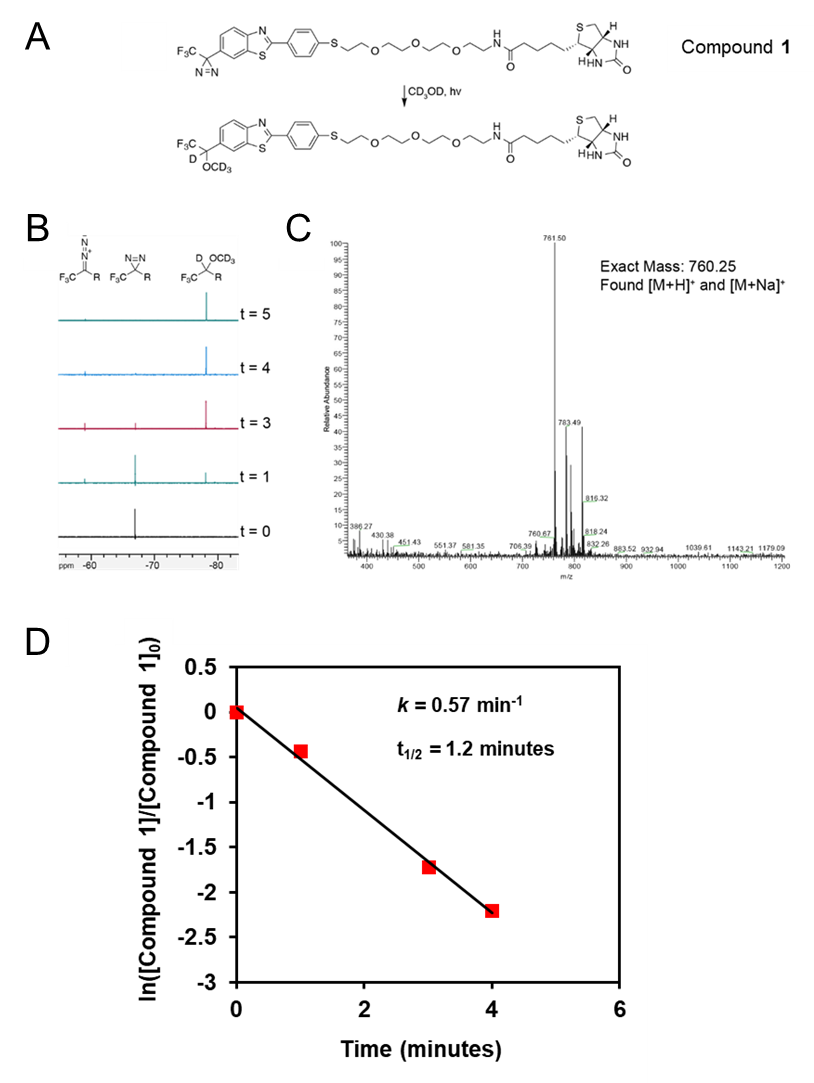


**Supplementary Figure S1. Photodecomposition Studies. A)** The reaction of compound **1** when exposed to UV light in deuterated methanol. **B)** ^19^F NMR study. Spectrum of reaction mixture is shown after 1 minute, 3 minutes, 4 minutes, and 5 minutes of irradiation in CD_3_OD. Reaction mixture was analyzed by ^19^F NMR and the disappearance of the signal from the trifluoromethyl group in compound **1** was monitored over time. Proposed corresponding structure is shown above each peak. **C)** ESI mass spectrum of the product of compound **1** irradiated in CD_3_OD shown in (A). **D)** Quantification of ^19^F NMR Data shown in (B) to determine rate of reaction. To determine the fraction of compound **1** remaining, the initial integration of the peak of compound **1** was compared to the integration of the same peak at a given time point. The natural logarithm of the fraction of compound **1** remaining was taken and was plotted against time. The data was fitted to the integrated first order rate law to determine kinetic parameters.


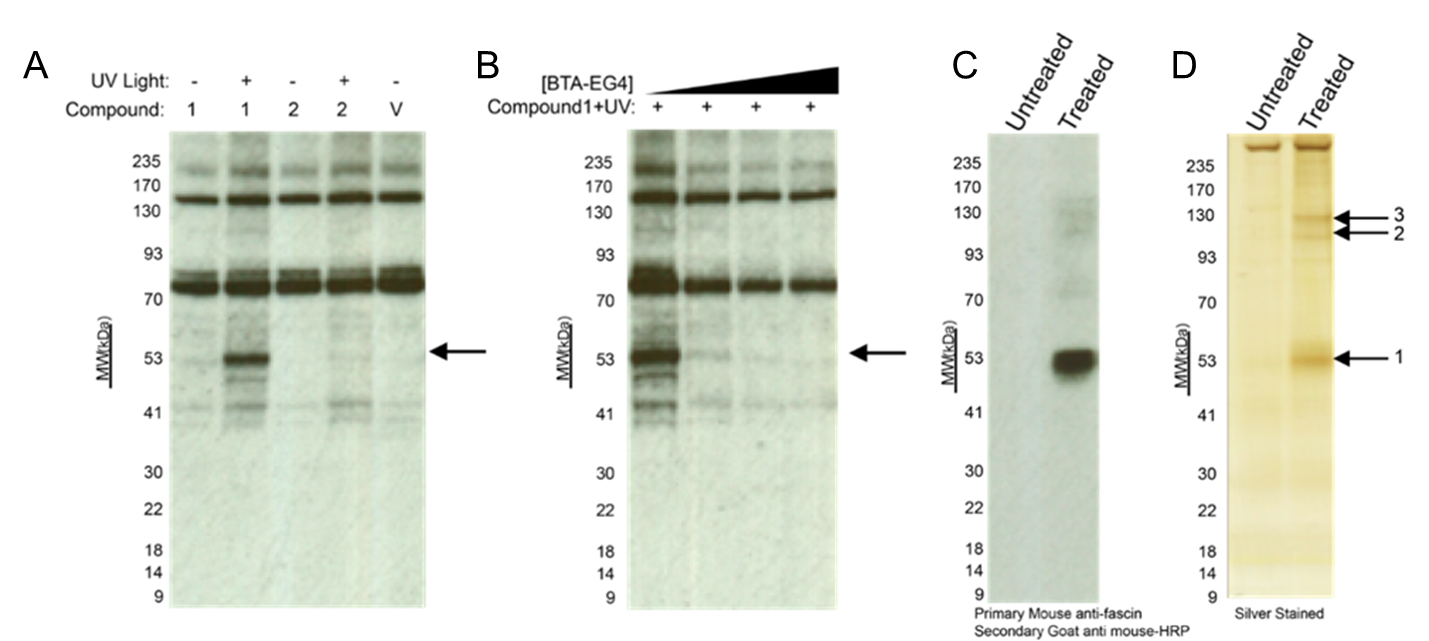


**Supplementary Figure S2. Western blot and SDS-PAGE analysis of human brain cortex lysates after various treatments. A)** Blot of cross-linking experiments in human brain cortex lysates using compounds **1** and **2** in the presence and absence of UV light for photoactivation. V = vehicle control (1% DMSO). **B)** Cross linking experiments using 5 µM compound **1** in human brain cortex lysate in the presence of increasing concentrations of BTA-EG_4_ (0-500 µM). Arrows denote the new strong ~53 kDa band found only upon treatment of human brain cortex lysates with compound **1** and UV light. Blots were visualized using a streptavidin-HRP conjugate; bands found across all samples are likely natively biotinylated. Cropped versions of Figure S2A and S2B are provided as main Figures 1B and 1C to highlight the main observations from these blots. **C)** Blot of SH-SY5Y neuroblastoma lysates treated with compound **1** and UV light (treated) or control (untreated, in the absence of compound **1** and UV), followed by pulldown using neutravidin agarose beads and visualized by Western blot using a mouse anti-fascin-1 (MAB3582) antibody. **D)** SDS-PAGE of SH-SY5Y lysate treated with compound **1** and UV light (treated) or control (untreated, in the absence of compound **1** and UV), followed by pulldown using neutravidin agarose beads and visualized by silver stain. The bands labeled 1, 2, and 3 were excised and examined by LC-MS/MS analysis (see table S1 for the results from the LC-MS/MS analysis of bands 1-3).


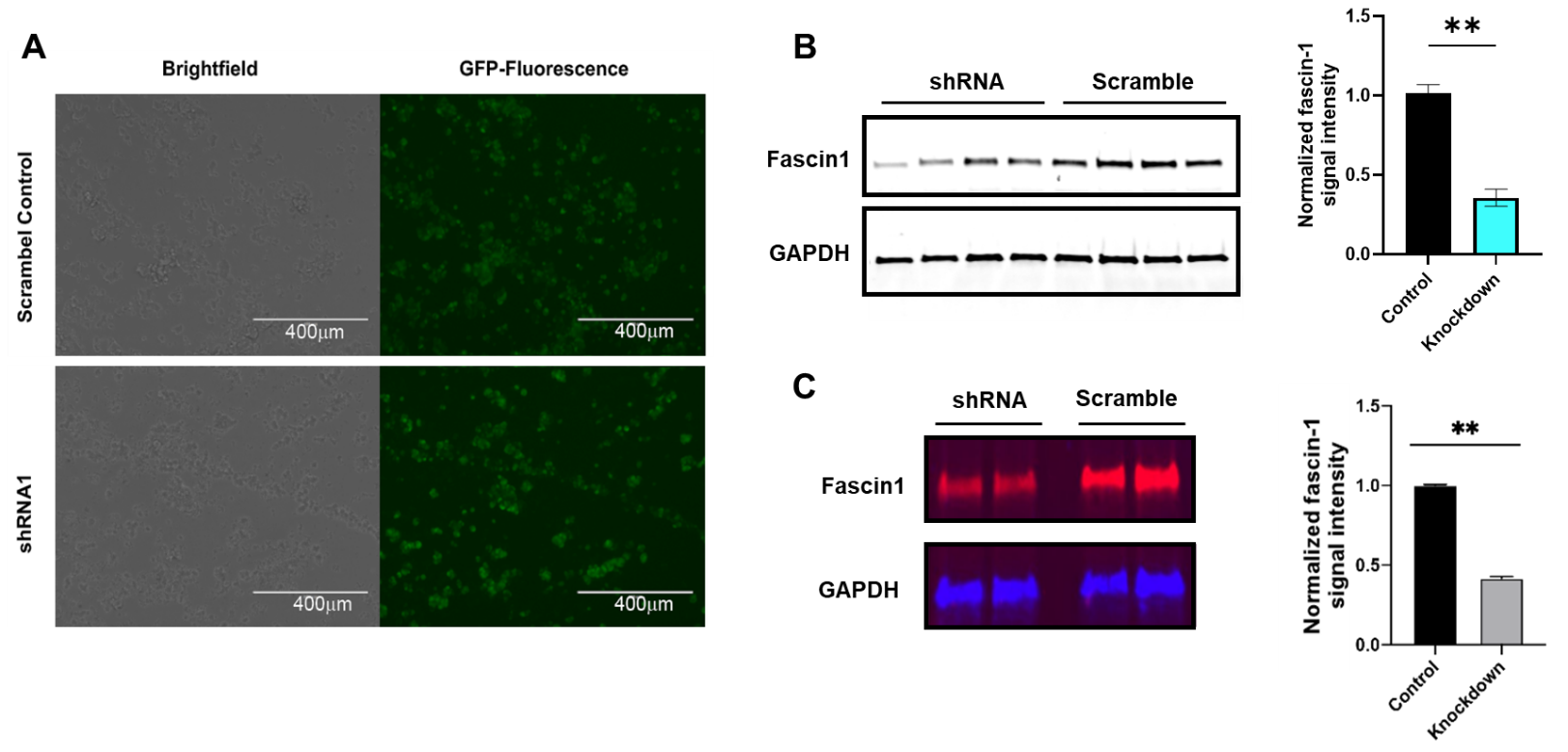


**Supplementary Figure S3. Verification of fascin-1 knockdown in PC12 cells and primary neurons.** **A)** Overall AAV infection levels of shRNA and scramble control verified by a GFP reporter gene in PC12 cells. **B)** Quantitative western blot and image analysis of fascin-1 expression levels relative to GAPDH control in PC12 cells treated with AAV plasmids. Cells treated with fascin-1 knockdown shRNA showed ~65% reduction in facsin-1 expression compared to the cells treated with scramble control **C)** Quantitative western blot and image analysis of fascin-1 expression levels relative to GAPDH control in primary neurons treated with AAV plasmids. Samples treated with fascin-1 knockdown shRNA showed ~60% reduction in fascin-1 expression compared to the scramble control. **p-value ≤ 0.01 as assessed by Student’s *t*-test.


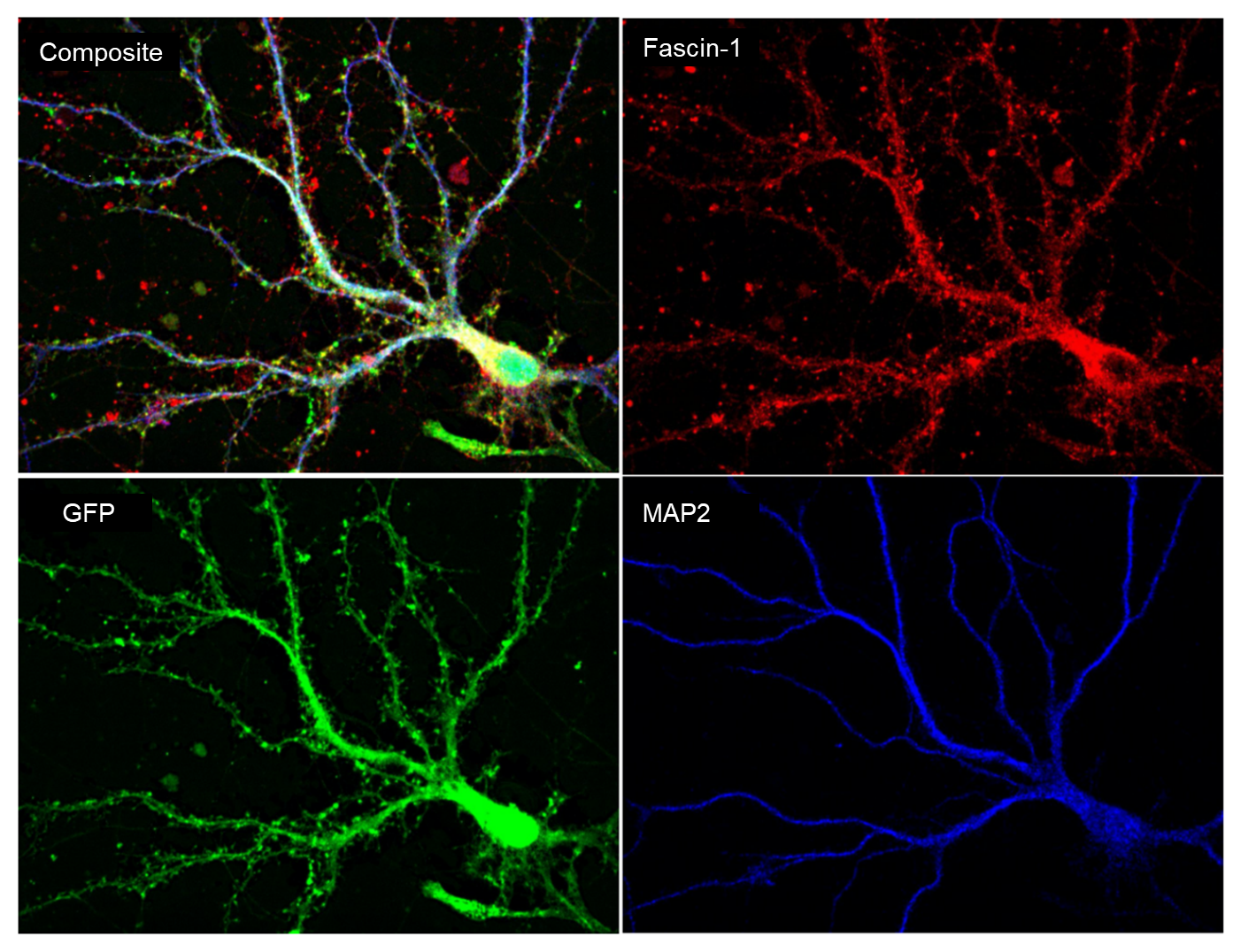


**Supplementary Figure S4. Immunofluorescent image of a mature primary rat neuron.** The neuron expresses a filler green fluorescent protein (GFP), stained for a microtubule associated protein used to identify dendrites (MAP2) and stained for fascin-1.


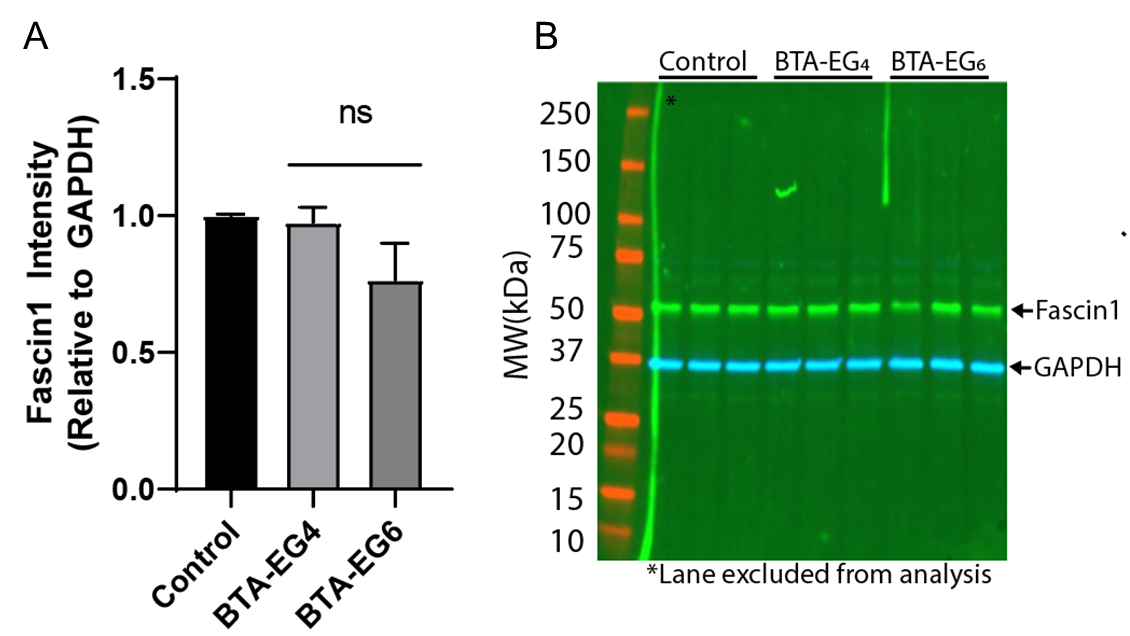


**Supplementary Figure S5.** **Fascin-1 expression levels in primary neurons treated with BTA molecules. A)** Quantification of fascin-1 expression levels in primary neurons treated with BTA-EG_4_ (5 µM) or BTA-EG_6_ (5 µM) compared to treatment with vehicle control (0.1% DMSO). **B)** Western blot with lysates from primary neurons treated with vehicle control (0.1% DMSO), BTA-EG_4_ (5 µM) or BTA-EG_6_ (5 µM). Each lane represents a technical repeat. Statistical analysis performed using two tailed Welch’s *t*-test.


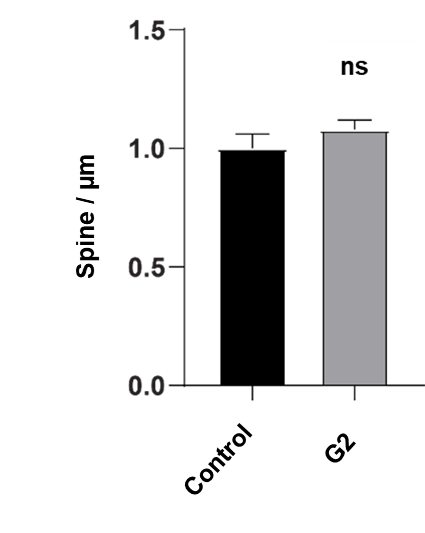


**Supplementary Figure S6.** **Spine density analysis of primary neurons treated with G2**. Spine density analysis of primary neurons treated with vehicle control (0.1% DMSO) or G2 (5 µM). Statistical analysis was performed using two tailed Welch’s *t*-test (n~35 dendritic segments per treatment).

**
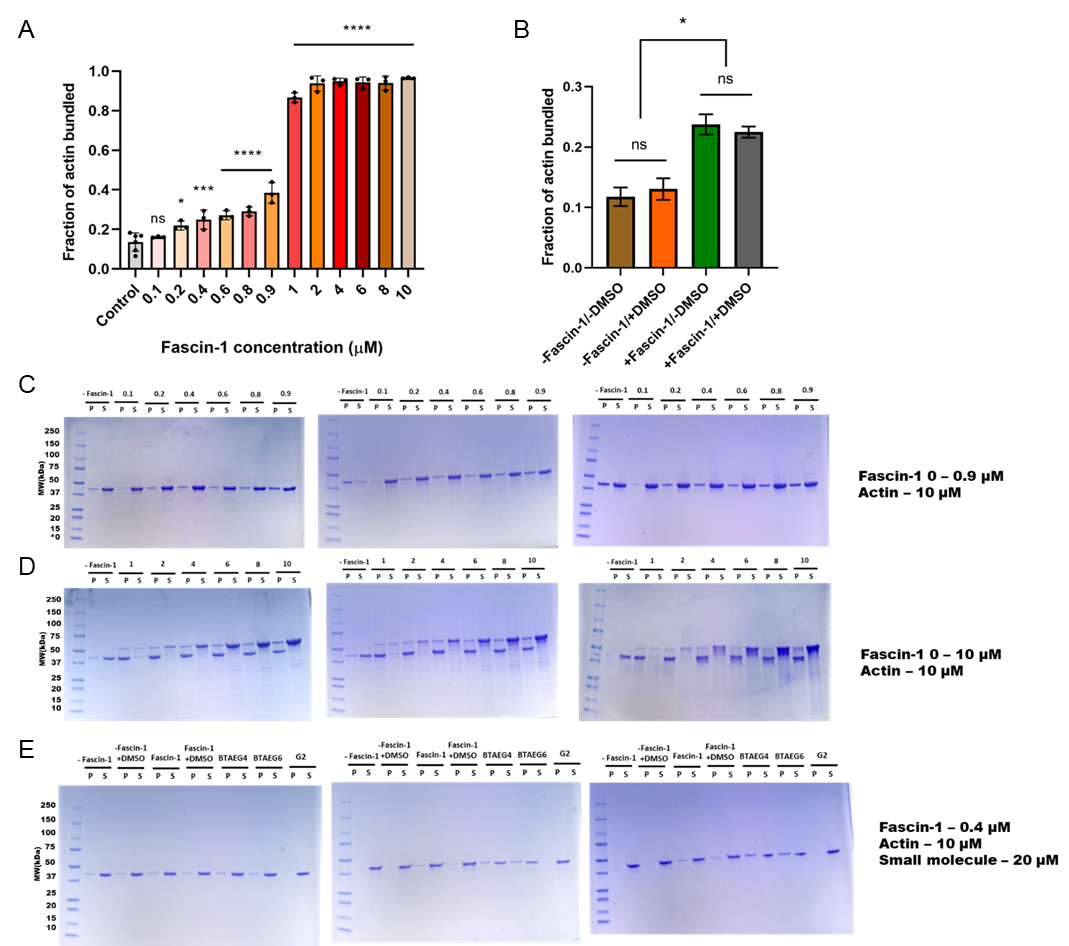
**

**Supplementary Figure S7. Data examining the actin bundling activity of fascin-1. A)** Graphical representation of the actin bundling activity of fascin-1 at 10 µM actin and varying concentrations of fascin-1. Statistical analysis done by one-way ANOVA (*p<0.05, ***p<0.001, ****p<0.0001) N=3 for each experiment. **B)** Graphical representation of actin bundling at 0.4 µM fascin-1 with and without vehicle control (1% DMSO). The addition of vehicle did not cause a significant change in actin bundling whereas fascin at 0.4 µM had a significant increase. Statistical analysis by two tailed Student’s *t*-test (*p<0.05). N=3 for each experiment. **C)** Replicate gels of actin bundling by fascin-1 (0-0.9 µM). **D)** Replicate gels of actin bundling by fascin (0-10 µM). **E)** Replicate gels of actin bundling by fascin at 0.4 µM in the presence/absence of small molecules (20µM) or vehicle controls. Each replicate in (C), (D), and (E) was performed using a separate batch of recombinantly expressed and purified fascin-1.


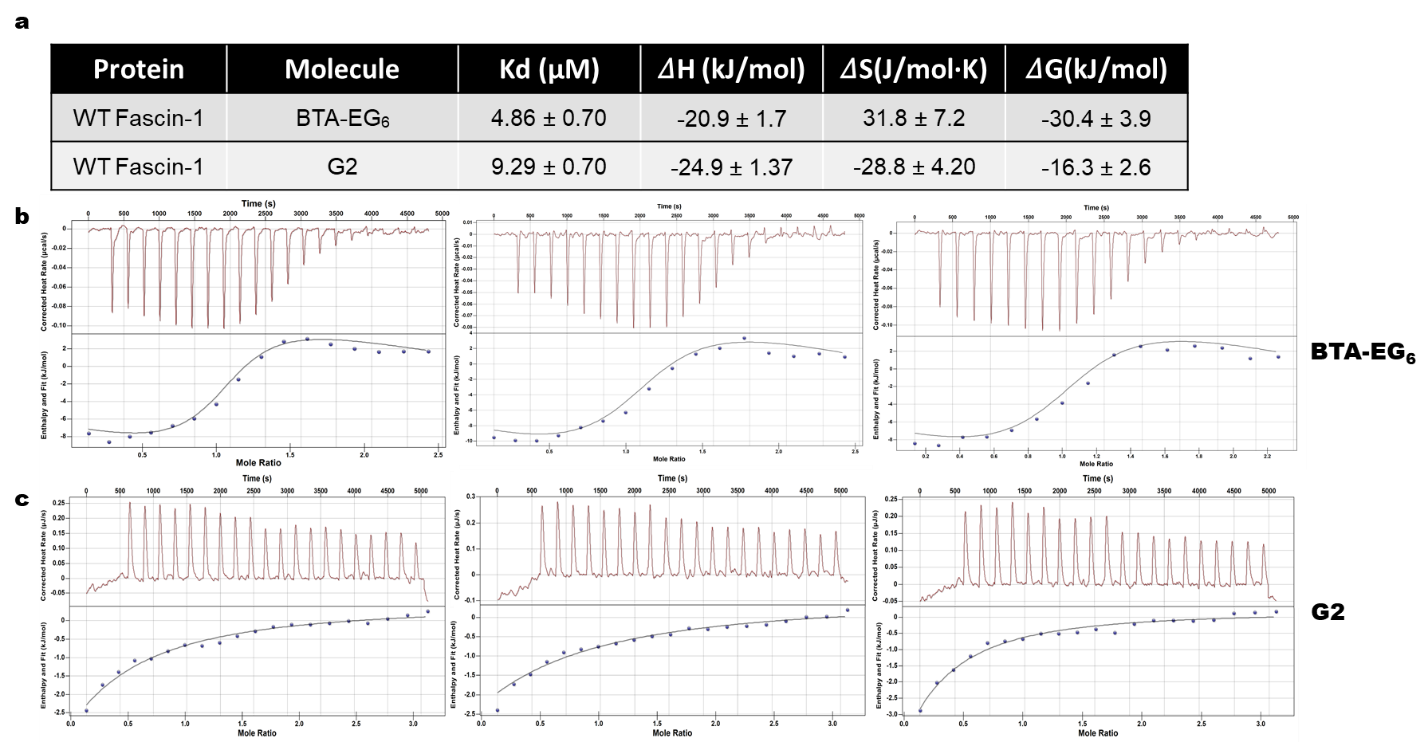


**Supplementary Figure S8. Results from ITC measurements of BTA-EG_6_ and G2 with WT fascin-1**. The experiments were carried out at 25˚C. The data represents measurements after subtraction of background (compound injected into buffer in the absence of fascin-1 protein). N=3 for each experiment.

**Supplementary Figure S9.** **Top docked poses of BTA-EG_6_ and G2 molecules bound to sites 1 and 2 on fascin-1.** BTA-EG_6_: At binding site 1, residues A137 and I45 form hydrophobic interactions while residues R389 and E492 form H-bond interactions. At binding site 2, BTA-EG_6_ forms hydrophobic interactions with residue I93, as well as cation-π and C-H-π interactions with residues R217 and E215, respectively. G2: At binding site 1, residues I45 and E492 are involved in hydrophobic and H-bond interactions respectively. At binding site 2, I93 forms hydrophobic interactions while residues E215 and R217 form H-bond and cation-π interactions respectively.


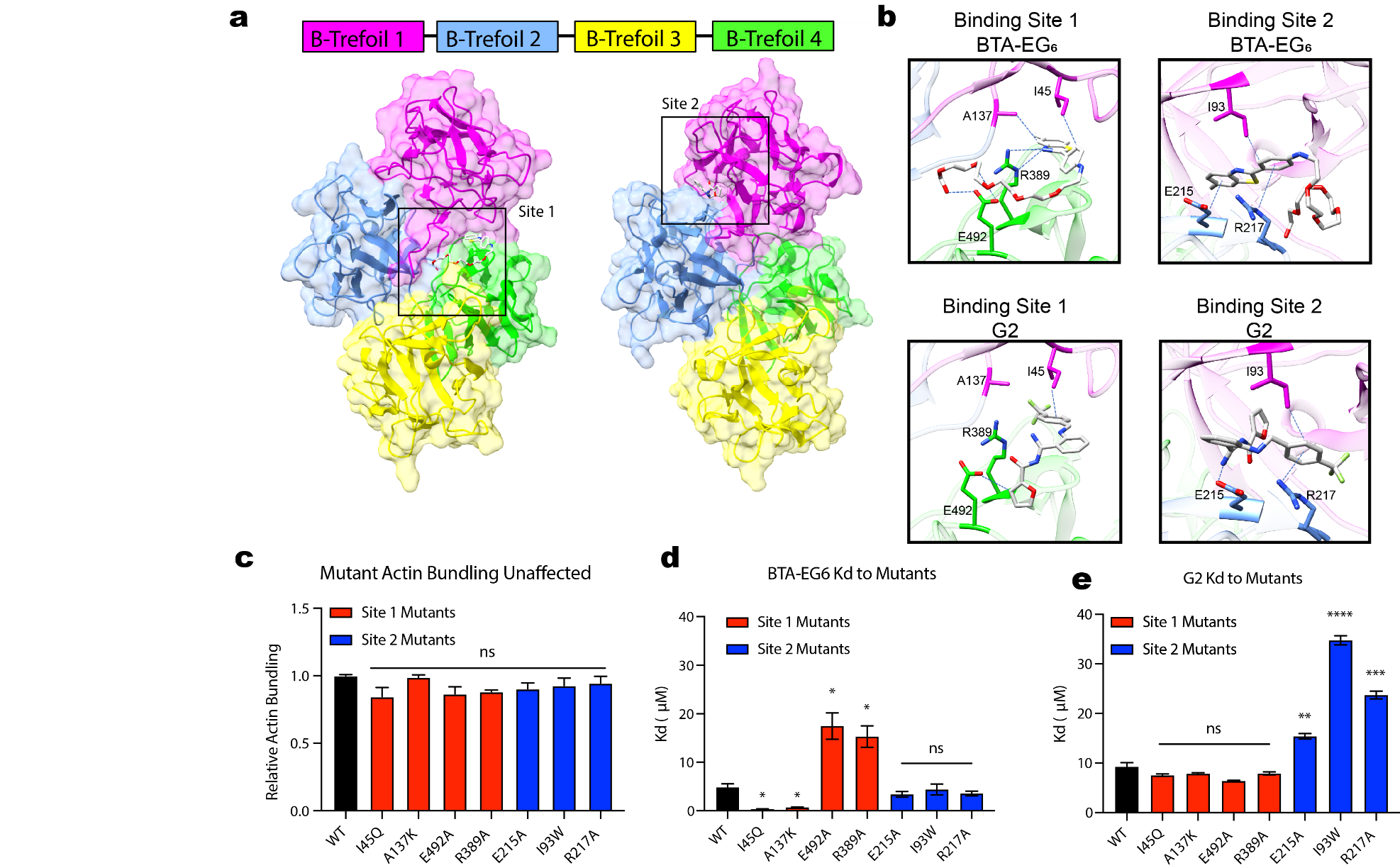

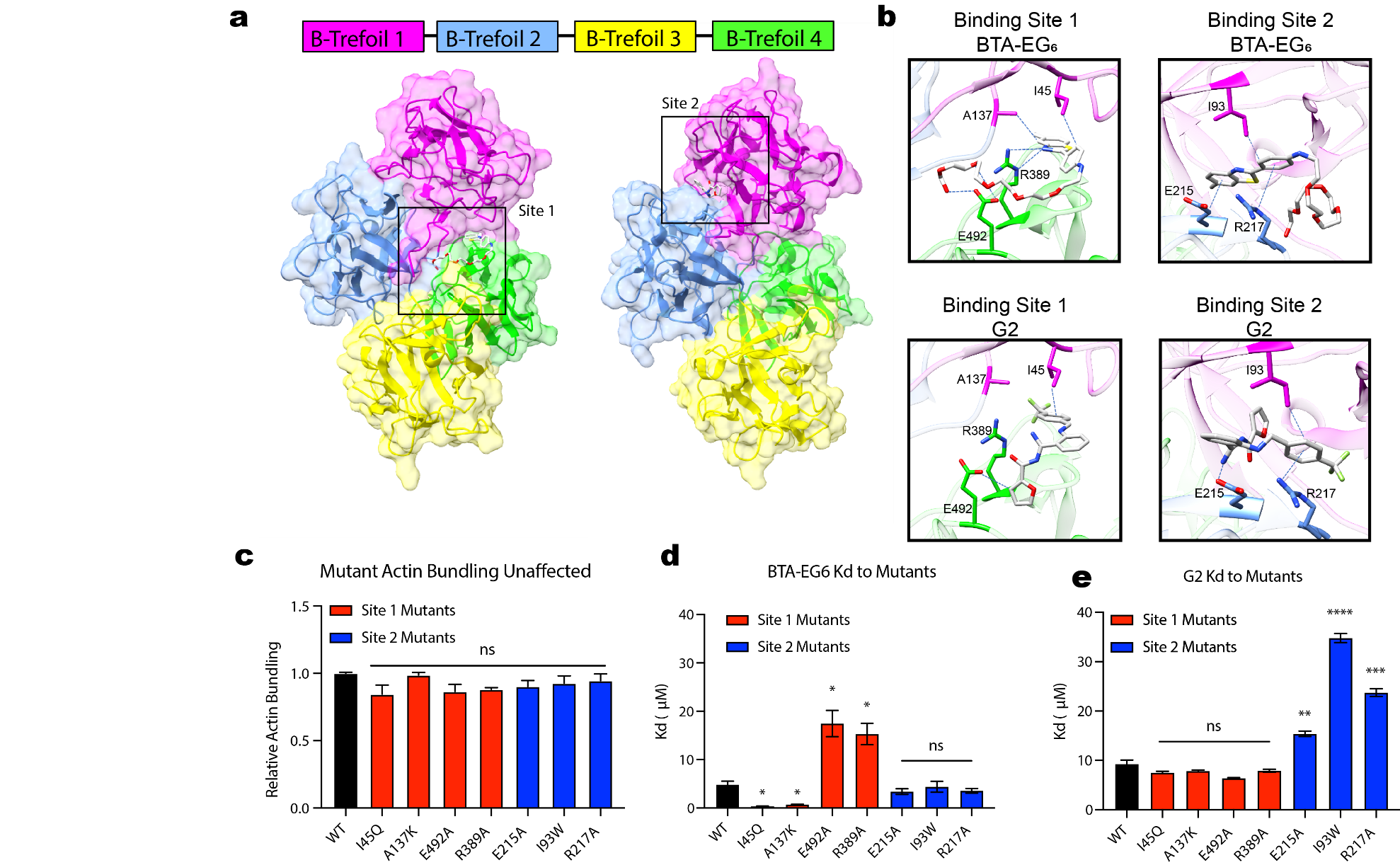

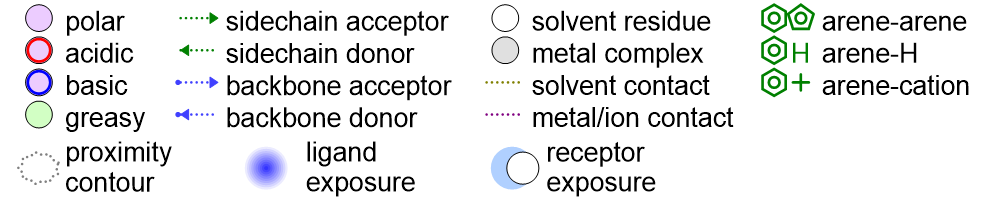

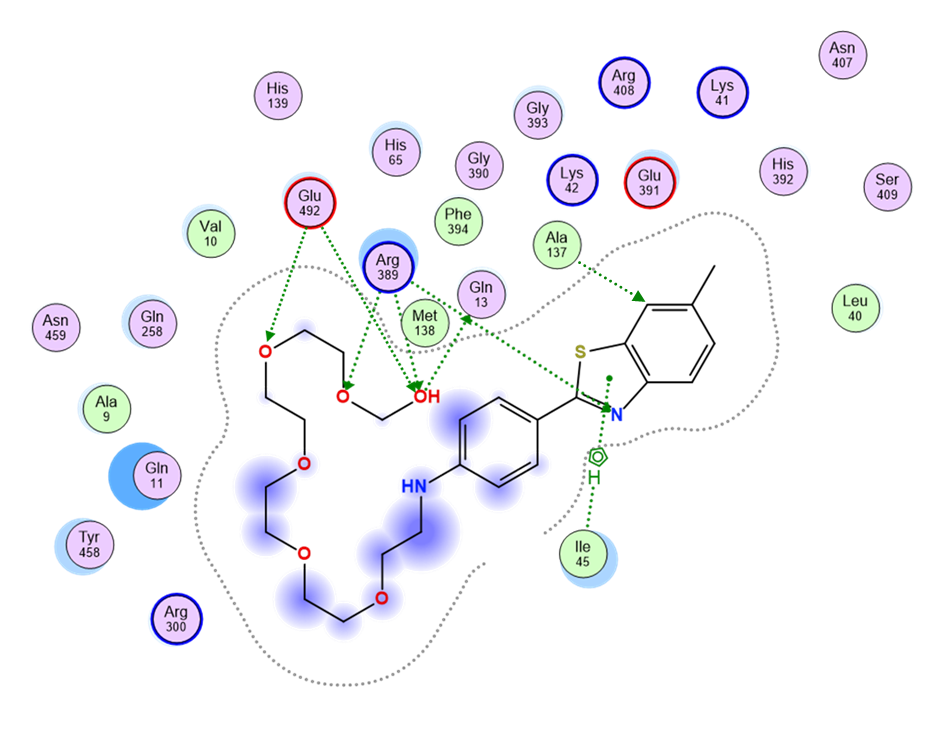


**Supplementary Figure S10.** 2D interactions map between fascin-1 and BTA-EG_6_ at binding site 1 between β-trefoils 1 and 4.


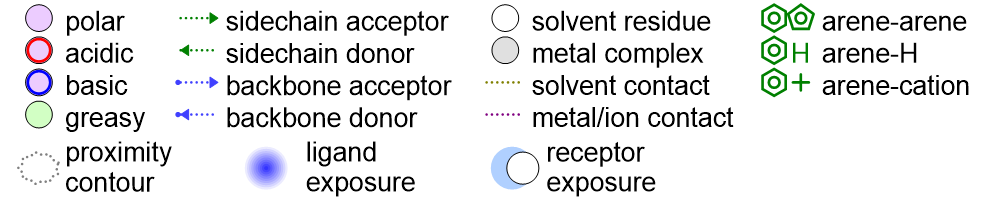

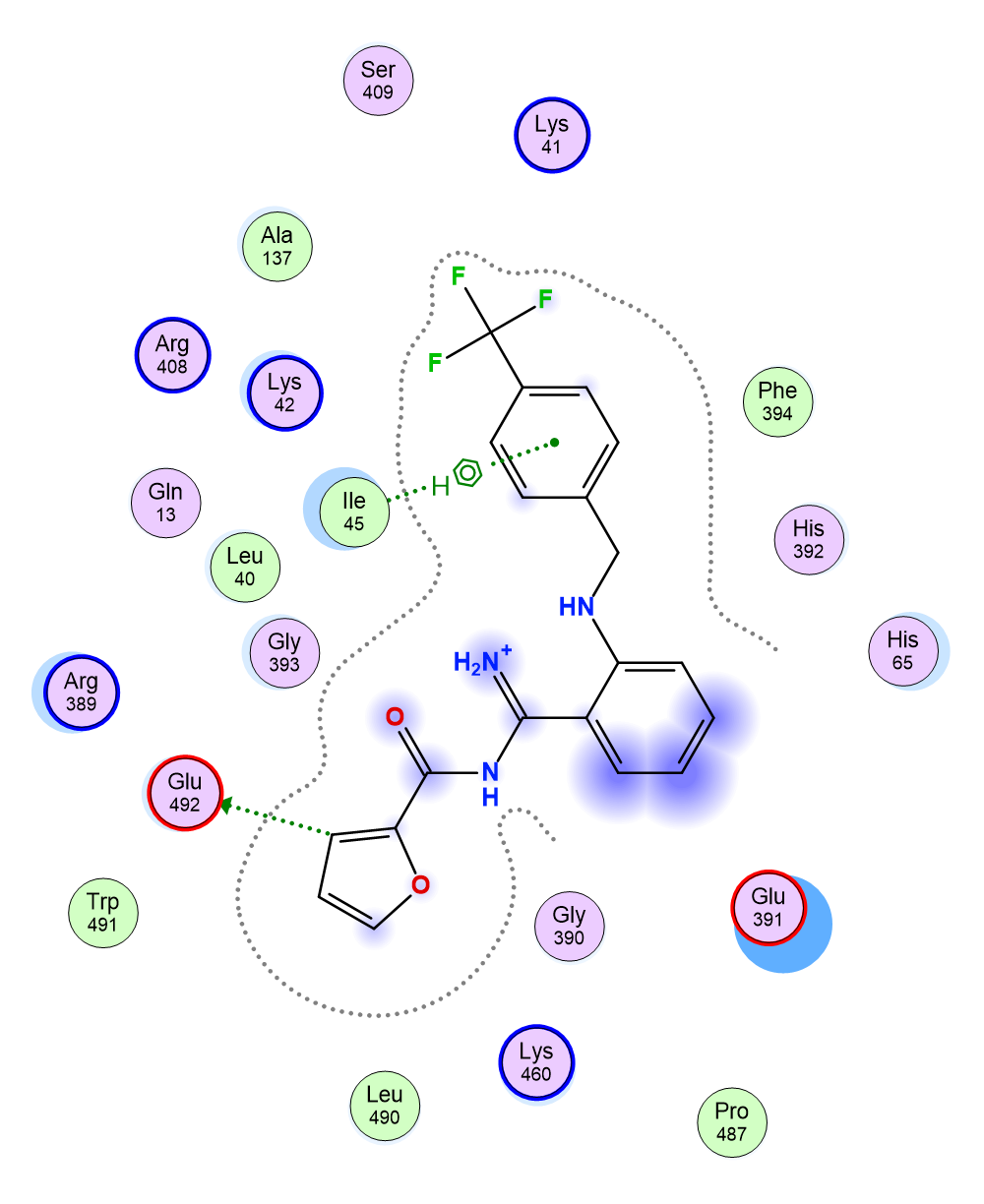


**Supplementary Figure S11.** 2D interactions map between fascin-1 and G2 at binding site 1 between β-trefoils 1 and 4.


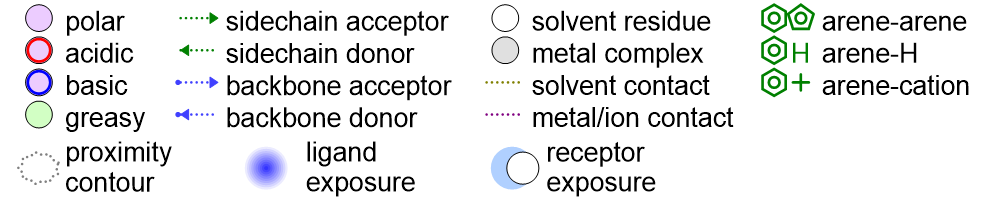

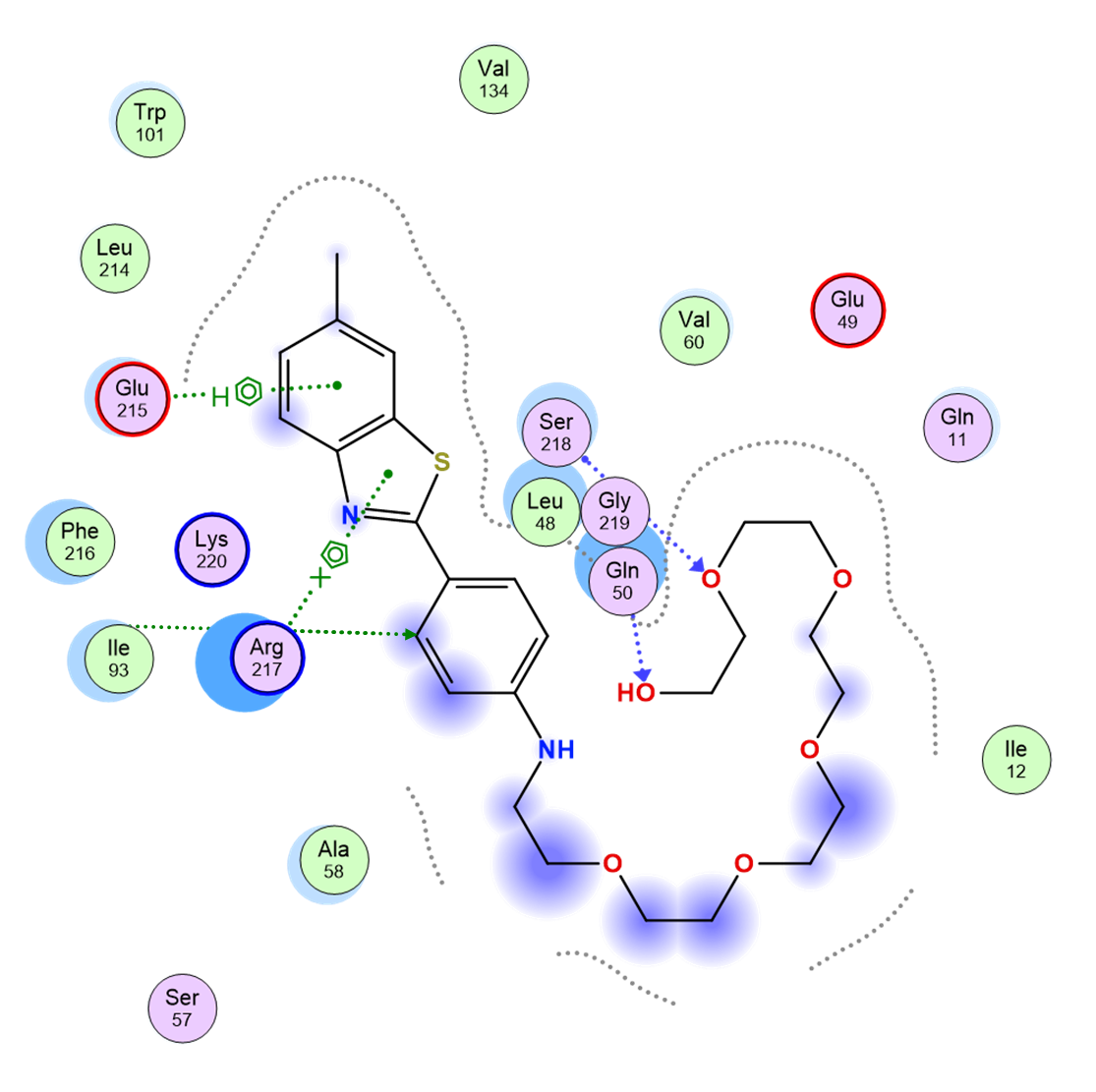


**Supplementary Figure S12.** 2D interactions map between fascin-1 and BTA-EG_6_ at binding site 2 between β-trefoils 1 and 2.

**Supplementary Figure S13.** 2D interactions map between fascin-1 and G2 at binding site 2 between β-trefoils 1 and 2.


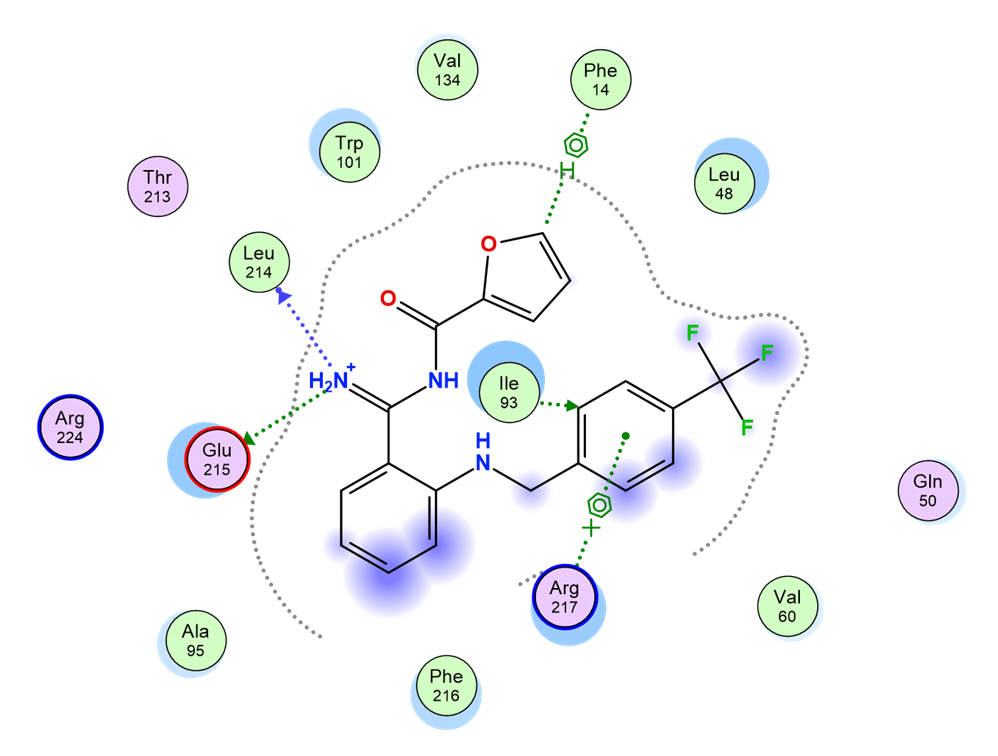

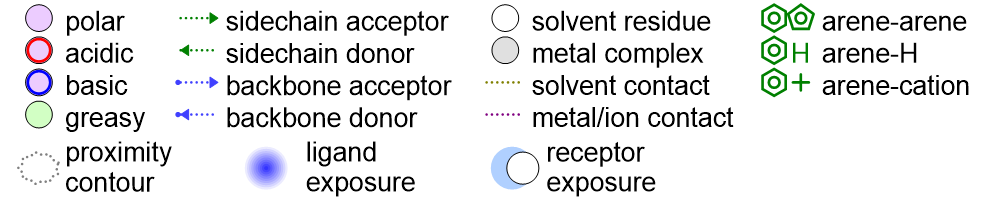


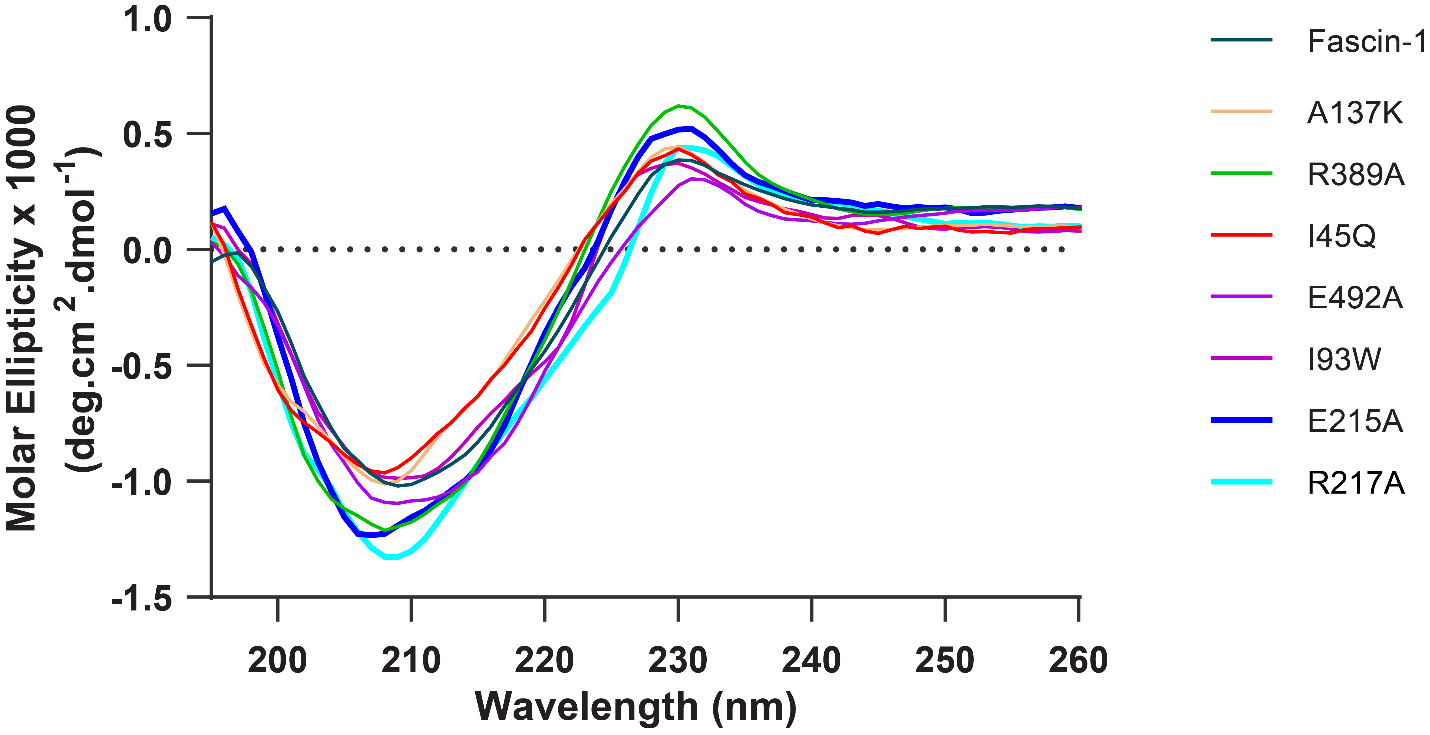


**Supplementary Figure S14. Circular dichroism spectra of recombinantly expressed WT fascin-1 and mutant fascin-1 proteins.** The shape of the spectra is consistent with large content of beta trefoil structure in fascin-1. The spectra of all the fascin-1 mutant proteins align well with that of WT fascin-1 with slight shifts. The activity was assessed using actin bundling sedimentation assay which showed all facin-1 mutant proteins were active and functional.


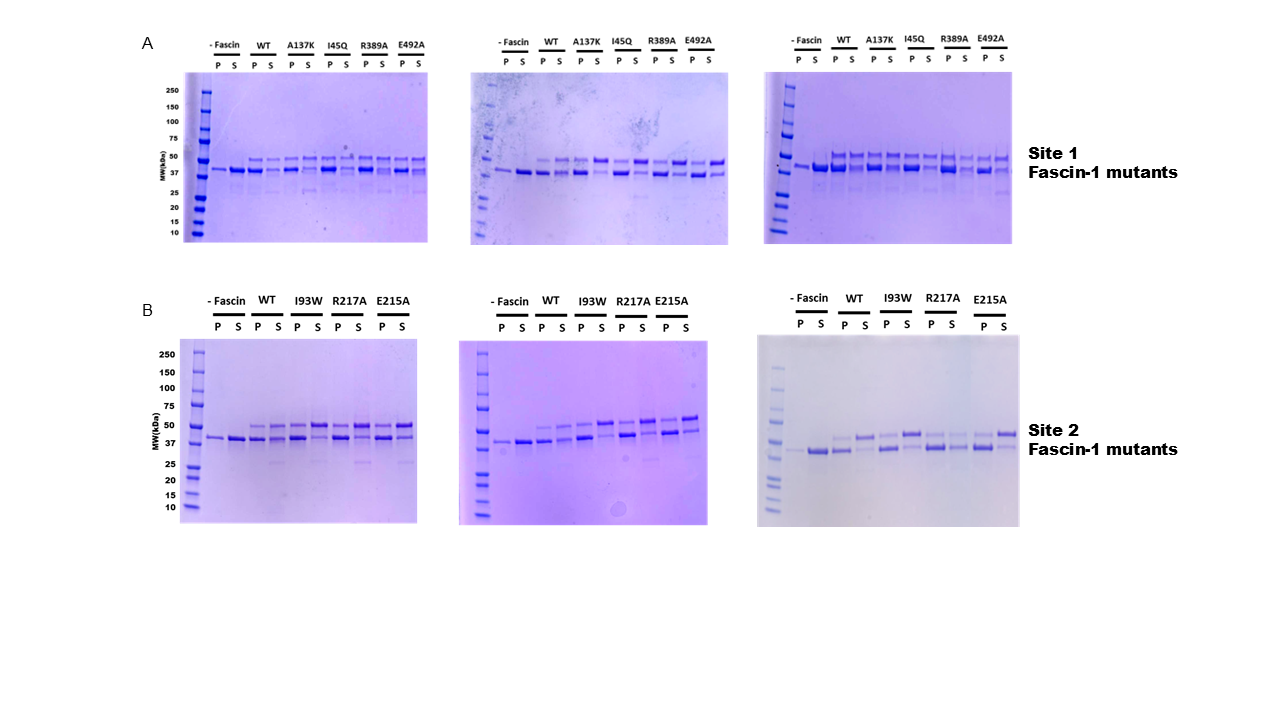


**Supplementary Figure S15. Technical replicates of the actin bundling activity of fascin-1 and fascin-1 mutants**. For all the experiments at site 1 (A) and site 2 (B), fascin-1 and actin concentrations were held constant at 10 µM.


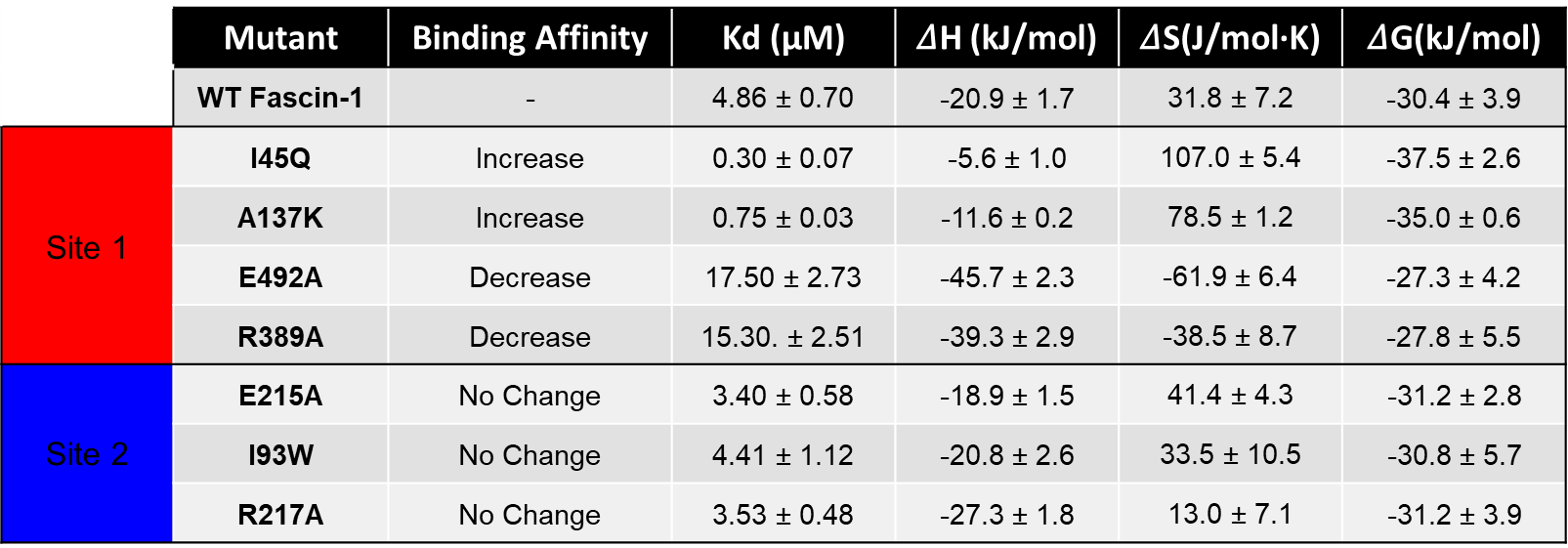


**Supplementary Figure S16. Results from ITC measurements of site 1 fascin-1 mutant proteins and BTA-EG_6_**. The experiments were carried out at 25˚C. The data represents measurements after subtraction of background (compound injected into buffer in the absence of fascin-1 protein). N=3 for each experiment.


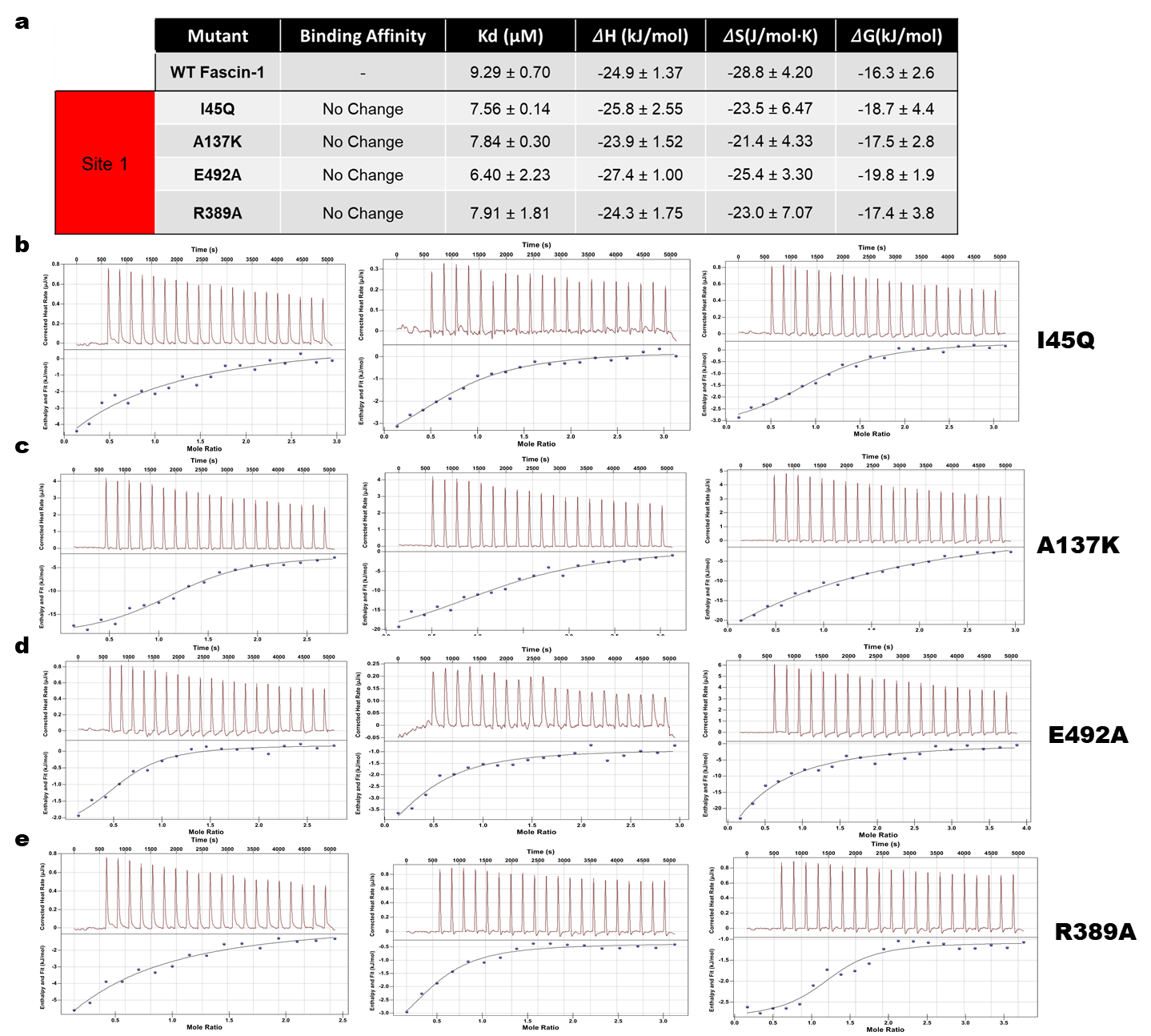
**Supplementary Figure S17. Results from ITC measurements of site 1 fascin-1 mutant proteins and G2.** The experiments were carried out at 25˚C. The data represents measurements after subtraction of background (compound injected into buffer in the absence of fascin-1 protein). N=3 for each experiment.


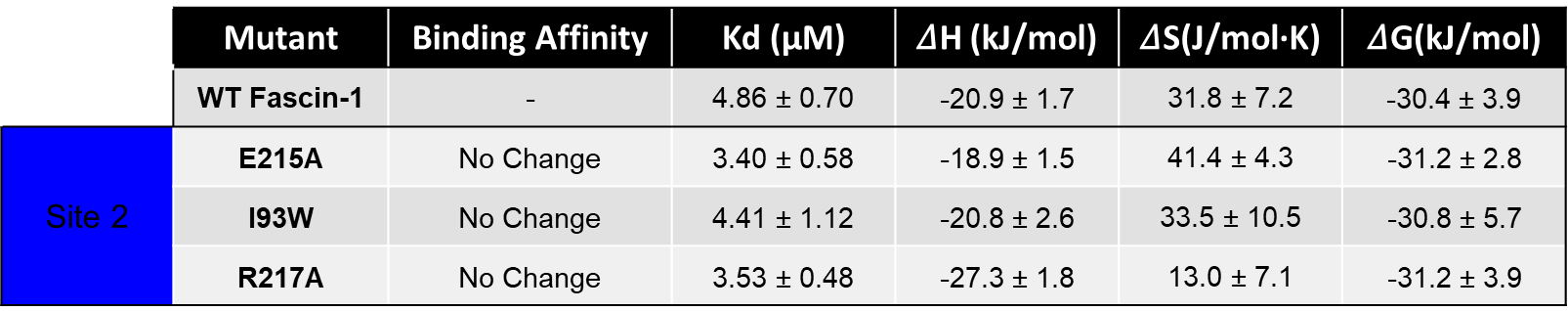


**Supplementary Figure S18. Results from ITC measurements of site 2 fascin-1 mutant proteins and BTA-EG_6_.** The experiments were carried out at 25˚C. The data represents measurements after subtraction of background (compound injected into buffer in the absence of fascin-1 protein). N=3 for each experiment.


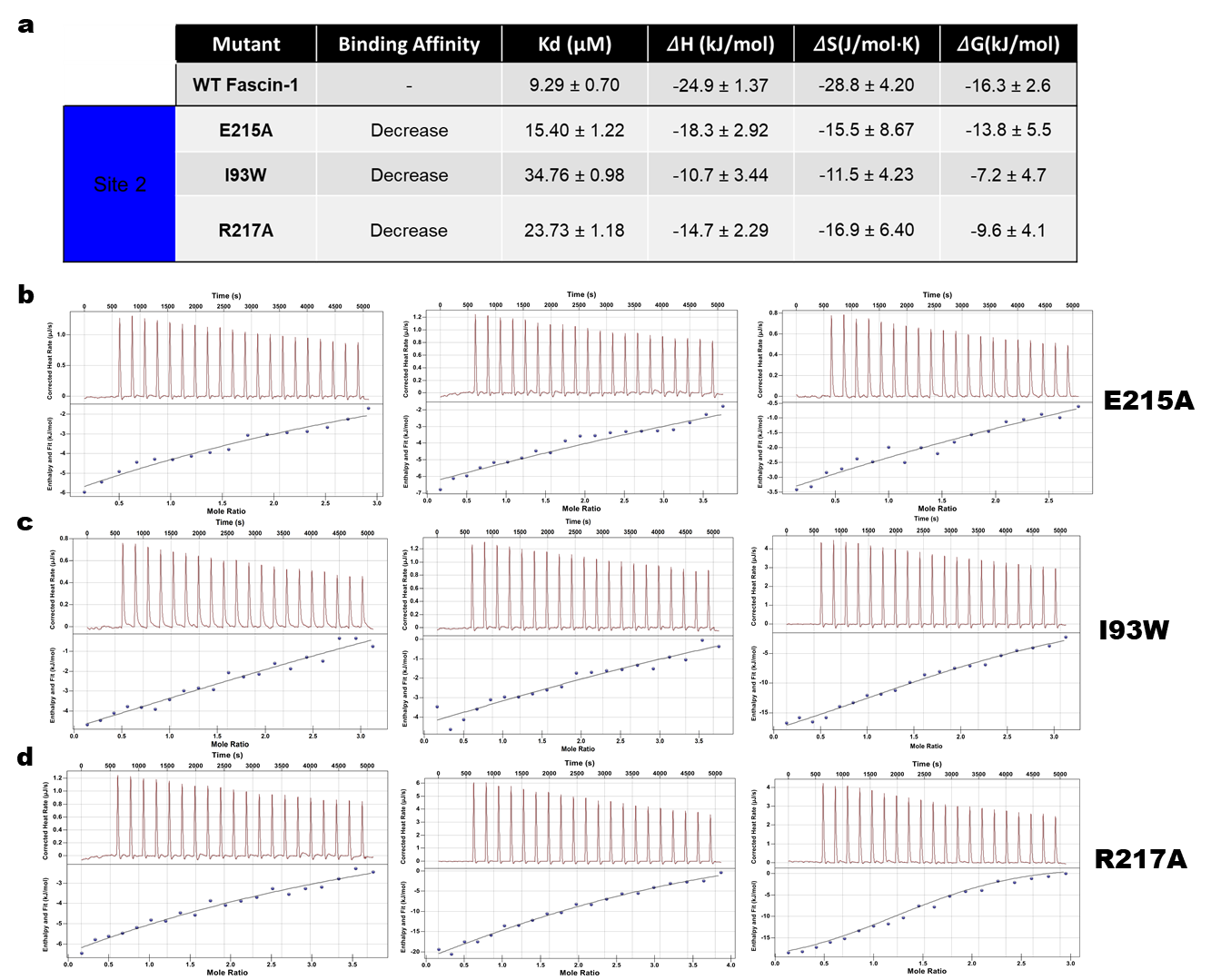


**Supplementary Figure S19. Results from ITC measurements of site 2 fascin-1 mutant proteins and G2.** The experiments were carried out at 25˚C. The data represents measurements after subtraction of background (compound injected into buffer in the absence of fascin-1 protein). N=3 for each experiment.


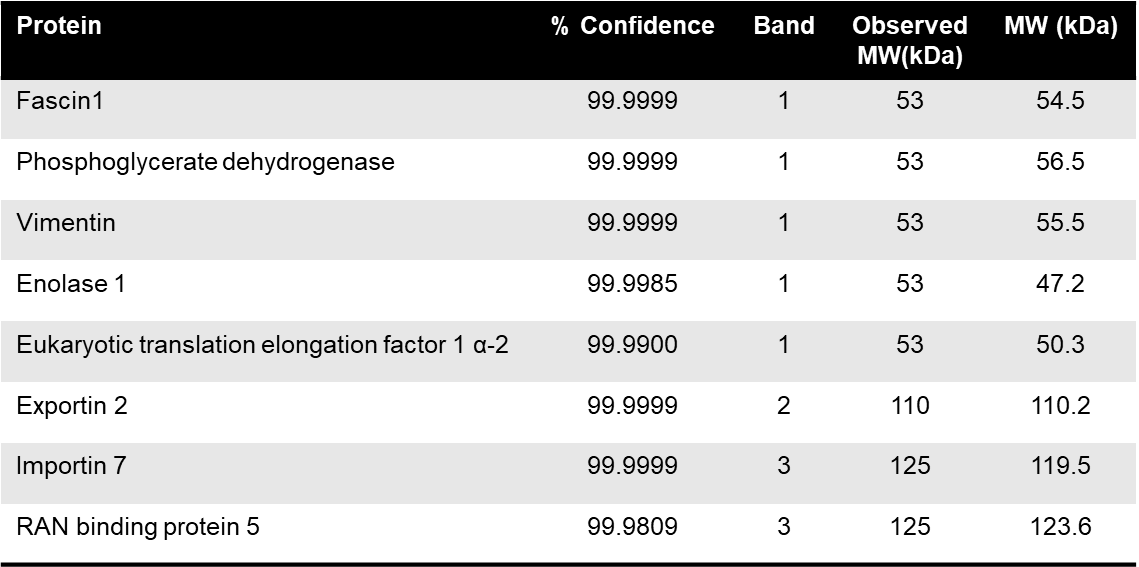


**Supplementary Table S1**. **Summary of results from LC-MS/MS experiments of excised bands in figure S2 (D).** Hits were included if they had a percent confidence of 95% or greater and were excluded if the hit was present in multiple bands, or if actual mass and apparent mass of the hit according to SDS-PAGE were inconsistent.

Data S1. (separate file)

Full list of proteins identified from the TMT analysis

**Supporting References**

68. Majek, M. & von Wangelin, A. J. Organocatalytic visible light mediated synthesis of aryl sulfides. *Chem. Commun.* **49**, 5507–5509 (2013).

69. Parmar, S., Pawar, S. P., Iyer, R. & Kalia, D. Aldehyde-mediated bioconjugation: Via in situ generated ylides. *Chem. Commun.* **55**, 14926–14929 (2019).
